# The role of dominance in sibling relationships: differences in interactive cooperative and competitive behavior

**DOI:** 10.1038/s41598-023-38936-7

**Published:** 2023-07-22

**Authors:** Lucia Hernandez-Pena, Wiebke Hoppe, Julia Koch, Charlotte Keeler, Rebecca Waller, Ute Habel, Rik Sijben, Lisa Wagels

**Affiliations:** 1 Department of Psychiatry, Psychotherapy and Psychosomatics, Faculty of Medicine, RWTH Aachen, Pauwelsstrasse 30, 52074 Aachen, Germany; 2 JARA - Translational Brain Medicine, Aachen, Germany; 3 Department of Psychology, University of Pennsylvania, Philadelphia, PA USA; 4 Institute of Neuroscience and Medicine: JARA-Institute Brain Structure Function Relationship (INM 10), Research Center Jülich, Jülich, Germany; 5 Brain Imaging Facility, Interdisciplinary Center for Clinical Research (IZKF), RWTH Aachen University, Aachen, Germany

**Keywords:** Human behaviour, Social behaviour

## Abstract

Siblings strongly influence each other in their social development and are a major source of support and conflict. Yet, studies are mostly observational, and little is known about how adult sibling relationships influence social behavior. Previous tasks exploring dynamically adjusting social interactions have limitations in the level of interactivity and naturalism of the interaction. To address these limitations, we created a cooperative tetris puzzle-solving task and an interactive version of the chicken game task. We validated these two tasks to study cooperative and competitive behavior in real-time interactions (N = 56). Based on a dominance questionnaire (DoPL), sibling pairs were clustered into pairs that were both low in dominance (n = 7), both high in dominance (n = 8), or one low and one high in dominance (n = 13). Consistent with our hypothesis, there were significantly more mutual defections, less use of turn-taking strategies, and a non-significant trend for reduced success in solving tetris puzzles together among high dominance pairs compared to both other pair types. High dominant pairs also had higher Machiavellian and hypercompetitiveness traits and more apathetic sibling relationships. Both tasks constitute powerful and reliable tools to study personality and relationship influences on real and natural social interactions by demonstrating the different cooperative and competitive dynamics between siblings.

## Introduction

Sibling relationships are highly significant within human social relationships. They are among the longest^1,2^ and exert a mutual influence on cognitive, emotional, social, and moral development^3^. In adulthood, sibling relationships become more symmetrical, egalitarian, supportive, and less competitive^4,5^. This increased harmony is partly explained by physical separation that typifies adult sibling relationships, which reduces the number of encounters and the salience of any prior rivalry^5^. Based on meta-analytic evidence^6^, greater sibling warmth, less differential treatment, and less conflict are associated with fewer internalizing and externalizing problems among both siblings.

Siblings can provide support, companionship and serve as confidants and role models^7^. However, they are also commonly a source of conflict and competition^8–10^. From an evolutionary perspective, conflict over limited resources drives sibling rivalry^11^. Sex (same-sex vs. opposite-sex siblings), birth spacing, degree of relatedness, and length of co-residence all contribute to determining the nature of the sibling relationship^11–13^. One of the most common sources of sibling conflict arises from relative power^11^, making dominance a major topic. Longitudinal data shows that older siblings tend to have a more dominant role, although this dynamic decreases during adolescence^4,14^.

Siblings remain of great importance in adulthood, especially for financial and emotional support^10,15^. However, only a limited number of studies have investigated sibling relationships in adults. Closeness between adult siblings is associated with positive affect, emotional maturation, and fewer conflicts and power struggles^16,17^. Importantly, sibling attachment in adulthood still significantly and uniquely predicts cooperation and conflict between siblings^18^. However, prior research on sibling relationships to date has been mostly observational^9,16,19^, relied on self-report questionnaires^11–13^, and largely focused on the perspective of a single individual^10,18^. To our knowledge, no experimental studies have explored the influence of dominance structures within sibling relationships in young adults during cooperative and competitive interactions.

Cooperation involves working together to achieve a common goal by solving a problem or making a decision^20^. In contrast, competition aims to outperform others to reach a desired outcome^21^. Cooperation and competition constitute models of interpersonal exchange, where individuals can either facilitate or hinder the achievement of the aims of another person^22^, so that person’s outcomes are dependent upon the other’s behavior ^23^. Previous studies have investigated cooperation using puzzle-solving tasks^24–28^. However, existing tasks have limitations, including turn-based actions and lack of complexity variation. A recent study circumventing some of these limitations used a tetris game where dyads played together in complementary roles to study verbal communication between players^29^. However, studies have yet to investigate how siblings play the tetris game together.

Another widely-used approach to study social interactions is based on the framework of game theory, mostly applying simulated scenarios using pre-programmed manipulated responses, or randomized strategies^27,30–34^. Well-known game theory tasks include social dilemmas, such as the prisoner's dilemma (PD)^35^, or the chicken game (CG)^30^ (also known as hawk-and-dove). The CG is a more realistic model than the PD because defection is the high-risk option, being more representative of real social life^31^. In these social dilemmas, the different outcomes for players are based on their free choice to cooperate or compete (defect).

To adequately study interpersonal exchange, it is essential to focus on both individuals within a dyad^32^. The willingness to cooperate or compete is influenced by the partner’s personality^33,34^. Interpersonal relationships in the context of games assessing cooperation and competition has been relatively understudied^9^. The limited existing research suggests that individuals tend to be more cooperative with friends compared to strangers, both in the CG or PD tasks^31^. No significant differences between friends and strangers were found when they played cooperative and competitive video games^36,37^. However, no prior studies have investigated how siblings play the CG task.

In addition to who plays the games, prior studies have investigated individual differences that influence social decisions. Personality traits that influence both cooperative and competitive behavior^38^ include hypercompetitiveness (i.e., need to compete and avoid losing at all costs)^39^, dominance (e.g., pursuit of power and coercing others)^40,41^, and Machiavellianism (i.e., focus on self-interest and personal gains at any cost)^42^. These personality traits are intercorrelated^41,43–45^, associated with lower cooperation in collaborative tasks^46,47^ and game theory tasks (for an overview see^38^), and linked to higher competitive and aggressive behavior in competitive contexts^48^.

Dominant individuals are those who are accustomed to “getting their own way” in social interactions and who prioritize their own gains, prevent other’s success and favor competition^49^. Submissive individuals are more likely to adopt a strategy of compromising when faced with a highly dominant partner^49^, and follow the “leader” in a cooperative task^50^. Cooperative partners may use a turn-taking strategy. Alternatingly turns allow the respective other to win points, and maximizing the possible points of both. To predict if siblings will cooperate, studies need to take account for the personality of each, as well as the type and quality of their relationship.

Our first goal was to study social interactions in a more immersive, dynamic, and interactive format. Thus, we created two tasks measuring different aspects of cooperation and competition to study the role of similarity in dominance, Machivellianism, and hypercompetitiveness in social interactions of siblings. Social interactions are defined as a set of social actions that are dynamically changing, with people able to adjust their choices based on the previous decision of their playing partner^51^. The new tasks necessarily involved: (a) two subjects engaged in the situation, (b) real-time interactions, (c) dynamic changes over time and (d) higher ecological validity compared to standard paradigms. Based on Hake's and Vukelich's^52^ system, our goal was to create and validate one task with *forced cooperation* to explore people’s behavior in situations in which cooperation represented the only possibility of obtaining the reinforcement, and a second task with *alternative choice* between cooperation and competition. Our second goal was to investigate the influence of individual and sibling differences on behavioral patterns within pairs, including (a) the participant’s personality, (b) the partner’s personality, and (c) the relationship between them.

## Hypotheses

We hypothesized that dominant individuals (high scores on the Dominance subscale of the DoPL) would score higher on competitiveness and Machiavellianism, as well as in the negative aspects of sibling relationships, such as apathy, competition, or criticism. We expected to find groups of pairs with either similar or dissimilar dominance (both low in dominance (LL), one low and one high in dominance (LH), and both high in dominance (HH) pairs).

In the cooperative tetris task, we expected that performance (defined as success in clearing lines together) a) would be better in easy compared to complex blocks, and b) would differ between pair groups (LL > LH > HH).

In the Interactive Chicken Game (ICG), we expected differences in dominant behavior, operationalized by the number of crashes, between pair groups (HH > LH > LL), and in cooperative behavior, measured by the number of turn-taking conditions, i.e., alternating turns to maximize both scores, (LL > LH > HH), and in the pair-related differences in their total ICG scores (HH = LL < LH), see Table S3 for further description of the measures.

For further details of all the hypothesis tested in the study, see the pre-registration in the Open Science Framework (OSF, https://doi.org/10.17605/OSF.IO/QDEVJ), and Supplementary Information (Sect. 1.1. Extended hypotheses).

## Methods

### Participants

The sample consisted of 28 same-sex sibling pairs, equaling a total of 56 participants (30 females, 26 males), with an average age of 23.39 (*SD* = 3.42) years. Due to technical problems, data for two pairs are missing for the Interactive Chicken Game task (one male and one female pair). For further sociodemographic characteristics of the participants see Table S7. Participants were recruited through flyers in University buildings, as well as on social media platforms. Inclusion criteria were same-sex sibling pairs aged 18–35 years, with a maximum age difference of five years (*M*_*diff*_ = 2.29, *SD* = 1.41) that minimized potential differences in stage of life or demographic characteristics. In addition, included siblings had to have lived together for a minimum of 10 years to control for relatedness and length of co-residence. Exclusion criteria were impaired vision, past or present neurological or psychiatric disorders, and a history of drug abuse. Each participant received a compensation of 30€ for study participation. The study was approved by the ethics committee of the Medical Faculty of RWTH Aachen University. All participants gave written informed consent in accordance with the Declaration of Helsinki^53^ prior to participation.

### Procedure

Data collection took place at the University Hospital Aachen in Germany. Upon arrival, sibling pairs received oral and written information and instructions, gave a first saliva sample (measuring cortisol and testosterone levels, not part of this manuscript), and were asked not to discuss strategies for the tasks.

Participants were then seated in front of a computer in separated rooms and played the *Cooperative tetris task*, the *Interactive Chicken Game*, and the *interactive Taylor Aggression Paradigm* in the same fixed order. The results from the last task are not part of this manuscript. After the tasks, participants filled in the self-report questionnaires, described in detail below in the section “Questionnaires”, which they accessed via the survey online tool *SoSci Survey.* The study lasted about two and a half hours.

### Tasks

Tasks were programmed using Psychopy (version 2020.2.10) (https://www.psychopy.org/) with language-adapted versions for sisters and brothers. Pygame (version 1.9.6) was used for the two-player tetris game inside the cooperation tetris task. Communication for the tasks was facilitated through a virtual server using the socket module in Python (version 3.6.6.), for further details on the programs' set-up see Supplementary Information (Sect. 2.2. Programs and set-up).

### Cooperative tetris task

The task is a modified version of the single-player puzzle game tetris. In the original version, players have to place the descending tetris pieces with different shapes within lines to complete them. Completed lines disappear, so that now unattached pieces at the bottom drop down as many lines as are cleared. The modified cooperative tetris task involved two participants solving pre-defined puzzle games interactively. Here, each participant has a specific role: one can only rotate the piece, while the other can only move it horizontally. Thus, placing the piece in the right position and orientation to fill the gaps and clear a line is only possible with effective cooperation, understanding, and non-verbal coordination. Participants' aim was to clear as many lines as possible. Player roles were randomly assigned at the beginning and reversed after each block. To move pieces horizontally, participants used the right and left keys of the keyboard. To rotate pieces clockwise, participants used the up key. For each player, the keys of the opposite role were blocked by the program.

For the cooperative tetris task, 282 trials were pre-rated by independent individuals (n = 16) for difficulty and field preference (i.e., most plausible solutions according to the rater’s opinion) and 96 trials were selected. For more details on the creation and evaluation of the trials, see Supplementary Information (Sects. 2.3 and 2.4). The final version consisted of four rounds, each containing six blocks. Every block showed a different pre-defined setting of pieces already placed at the bottom of the squared block. One block comprised four trials, i.e., four new pieces. The four rounds were repeated with reversed player roles (rotating/moving horizontally), resulting in 192 trials.

**Figure 1:**
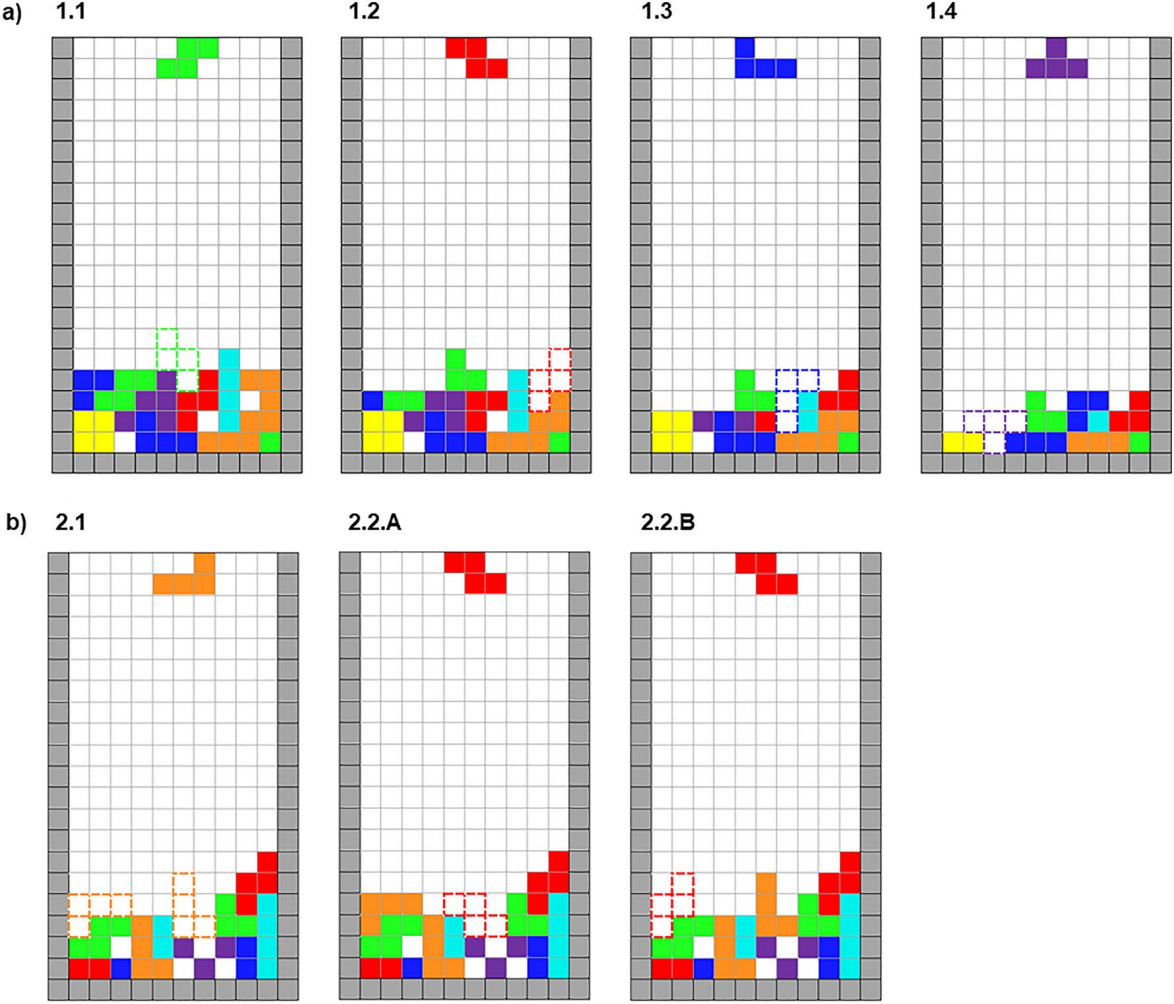
Representation of the cooperative tetris task. (**a**) Four trials in the easy block and a representation of the optimal solution (dashed lines). (**b**) First two trials of a complex block with a representation of two potential and equally advantageous positions for the piece in the first trial (2.1), indicated by orange dashed lines. Depending on which of these positions was chosen, the second trial was presented to the participant either as version 2.2A or 2.2B. The red dashed lines indicate the ideal position for the piece in the second trial. The other two trials of the complex block (shown in Supplementary Fig. S2) continue with two ideal positions for the next two pieces.

The blocks differed in their level of difficulty. Easy blocks (see Fig. 1a) had one particular position for the new piece that could immediately complete at least one line (odd numbered blocks). Therefore, participants could at least clear four lines at the end of the block (sometimes two lines could be cleared together).

Complex blocks (equal blocks, see Fig. 1b) were aimed at increasing conflict between the two players since there were two equally good positions for the first piece (unknown to the participants, both were actually suitable for the first and second pieces). If the first piece was placed strategically in one of the gaps, siblings could complete a line with the following piece. However, due to more ambiguous options, it was more difficult to cooperate during complex blocks. Participants could at least clear one line after the second piece and one after fourth piece, for a total of at least two lines per block (sometimes two lines could be cleared together). Success during performance was measured as number of cleared lines. During and at the end of the task, several outcome measures assessing performance and questions about their experience were collected (see Table S3 and Table S4).

### Interactive chicken game

**Figure 2:**
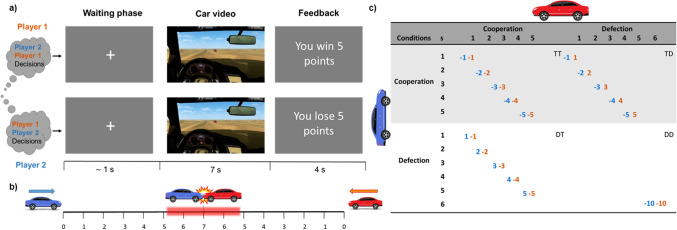
Representation of the Interactive Chicken Game task. (**a**) ICG trial showing the waiting phase that lasts until the connection with the server is confirmed. The car video phase includes a video with the driver's first-person perspective driving towards another car. This phase includes a decision phase until one or both participants decide to turn, or the point of no return is reached, and then the feedback video is shown depending on the outcome. The image shown in this figure is a similar representation of our actual video shot (not a screenshot); a feedback phase based on the decision of both players (according to the payoff matrix). (**b**) The numbers depict the different intervals in which participants can turn (each interval represents one second) equaling the size of the loss/gain, up to 5 s in which a point of no return is reached and a collision occurs. (**c**) The ICG payoff matrix*.* The numbers located on the outside of the table represent the seconds throughout the trial, while the numbers inside the table represent the points won or lost by each participant, which are equivalent to the waiting time to turn. ICG structure matrix is summarized as participant defects + other turns > both turn > turns + other defects > both defect (DT > TT > TD > DD, although when the participant is in the TT and TD conditions, the player loses the same points, in TD the other gains points, which puts the other player at an advantage against the total score).

This task is a modified version of the theoretical CG. In the original task, two players drive cars towards each other, the player turning loses and if none of them swerve, there is a car crash. The outcome of the CG is determined by both players’ decisions. From one person’s perspective, unilateral defection and mutual cooperation represent the best and second-best outcomes. Cooperation is preferable to defection if the other defects because mutual defection is worse than the benefit for unilateral cooperation, each payoff corresponding to different outcomes of the social interaction^23,54^. In contrast to the original CG, participants playing the Interactive Chicken Game did not decide between two options (cooperate or defect). They adopted a first-person driver’s perspective in the video of their car approaching the other player’s car and decided if and when they wanted to turn (see Fig. 2a). The videos of the approaching cars were created using the free online game *madalin stunt cars 2*, and included standard level audio for all participants with the sound of the accelerating engine as well as the sound of the car crash to increase immersion. In addition to improved immersion and interactivity, the Interactive Chicken Game continuously offered the option to turn (cooperate) while the risk of a crash increased as the trial progressed (see Fig. 2b). This measure of continuous choice allowed the analysis of different degrees of cooperation, which increased the strength of the effect in comparison to analyzing binary decisions^54^*.* The choice period was divided into five intervals of one second so that participants could turn at the same interval.

In each of a total of 42 trials, divided into three blocks, participants could decide either a) to defect, i.e., not to press a button and drive through, or b) to cooperate, i.e., pressing the “space” key to turn. The aim was to receive as many points as possible. If both players went straight (defect), their cars crashed (DD), losing 10 points. If both turned their cars at the same interval (TT), both lost the same number of points. If one player chose to drive through (defect) and the other player turned, the defector received points (winner, DT), while the cooperator lost points (loser, TD). In these cases, the number of points the players lost or received depended on the time at which participants decided to turn (see Fig. 2c for further details). Therefore, during the decision phase, each car video had different endings depending on the behavior of both participants: if the participant turned, the screen showed the video of the player's car turning at different intervals (1-5 s). If the other participant turned, the current player saw the other car turning at different intervals (1-5 s). If neither of them turned, they saw a car crash. Additionally, there was a “point of no return” after which it was no longer possible for the player to turn, and a crash became inevitable (after 5 s), see Fig. 2b.

Some outcome measures were based on response time (RT) defined as the time from starting the car until the space button was pressed to turn the car. RT was used to calculate the trial feedback and total IGC score, i.e., sum of feedback scores. Additionally, a sum of the decision combinations (both turn; TT, one-turning; DT + TD, both defect; DD) of each pair was calculated, see Table S3. After each block and at the end of the task, participants answered questions related to their experience and motivations during the task (see Table S5).

In addition, participants were informed beforehand that they could receive higher monetary compensation (up to 10 Euros extra) depending on which of the two players obtained more points in the task (and in the third task of the study). We instructed the participants that we would randomly take the result of one block of each task to compare the points. The purpose was to increase their motivation about getting points and winning the games. For ethical reasons and for partially manipulating the third task, all participants received the maximum amount of money (30 Euros).

### Questionnaires

Siblings answered questions about their relationship (Sibling type questionnaire, STQ; Stewart et al.^55^), assessing *Mutuality*, *Competition*, *Criticism*, *Apathy*, and *Longing*. To assess dominance traits, we used the *Dominance, Prestige, and Leadership Scale* (DoPL)^41^, with the following subscales: Dominance; DoPL-D, Prestige; DoPL-P, Leadership; DoPL-L. In order to measure competitiveness traits, the *Personal Development Competitive Attitude Scale* (PDCA)^56^ and *Hypercompetitive Attitude Scale* (HCA)^39^ were assessed. The *Machiavellianism Scale* (Mach-IV)^57^ was administered to measure Machiavellianism traits. To explore their relationship with others, *Personal Sense of Power Scale* (PSPS)^58^ was administrated and the *Rank Style With Peers Questionnaire* (RSPQ)^59^ was assessed to measure individuals’ social rank style, with the following subscales (RSPQ) *Dominant Leadership* (DL), *Coalition Building* (CB), and *Ruthless Self-Advancement.* For a more detailed description of the questionnaires, details on the subscales and reliability measures, see the Supplementary Information (Sect. 2.7).

### Statistical analysis

All analyses were performed using Matlab (v2017b, MathWorks, Natick, MA) and SPSS (IBM SPSS Statistics 20.0, Chicago, IL). We assumed a two-tailed level of significance of *α* = *0.05* for all analyses. Post-hoc analyses were corrected for multiple comparisons using the Bonferroni method. Shapiro–Wilk test statistics were applied to check for the assumption of normality of all outcome variables, and Levene’s test to check for the assumption of homogeneity of variances. Mutual Cooperation (both turn), total ICG score, and PSPS questionnaire scores did not meet the assumption of normality, therefore Kruskal–Wallis tests were used. Mutuality (STQ) and RSA subscale (RSPQ) scores did not meet the assumption of homogeneity of variances, thus Welch test, and Games-Howell tests for post-hoc pairwise analyses were applied.

### Cluster analysis

We adopted agglomerative hierarchical cluster analysis to identify distinct subtypes of sibling pairs. Cluster analyses are hypothesis-free methods and do not make initial assumptions about group characteristics with the aim to group together participants with similar patterns on specific clustering variables. We used Ward´s clustering method^60^ with squared Euclidean distance. Ward´s method categorizes individuals into clusters based on input variables and aims at minimizing within-cluster variance. Input variables were the sum of the individual DoPL-D scores and the absolute difference between the DoPL-D scores of the individuals in the pair. The cluster analysis generates a dendrogram for the estimation of the number of likely clusters and clusters were determined by cutting at the largest distance increase.

Demographic variables of the identified clusters were compared using the Chi-square test for categorical variables (sex, education, occupation, marital status), and one-way ANOVA for continuous variables (age, and age difference). To study the relationship between the DoPL-D and other questionnaire data (competition, criticism, and apathy subscales of the STQ, DoPL-P, DoPL-L, Mach-IV, HCA, PDCA, and RSRQ), Pearson correlation analyses were performed. Spearman correlation was calculated to investigate the relationship between DoPL-D and PSPS. The p-values of the correlations were corrected by applying Bonferroni correction (corrected α = 0.004). Moreover, we performed a one-way ANOVA with cluster as factor and the different sum and mean scores of pairs of the above-mentioned questionnaires as dependent variables to explore whether the pairwise sibling clusters differed on the different questionnaire measures.

### Cooperative tetris task analyses

To validate the task, a repeated-measures ANOVA was conducted with block difficulty (2 levels: easy and complex) as a within-subjects factor, cluster as a between-subject factor and number of cleared lines as dependent variable. Moreover, in order to study whether the different clusters differ in success, a one-way ANOVA was performed to compare the effect of cluster on the number of cleared lines per block. For exploratory analyses, see Supplementary Information (Sect. 2.8. Complementary analyses).

### Interactive chicken game analyses

To study group differences, a one-way between-subjects ANOVA was conducted to compare the effect of group on mutual defection, mutual cooperation, one-turning, and turn-taking conditions, as well as, pair total ICG score, pair difference in total ICG score and pair dominance ICG score, see Table S3 for further description of the measures. For exploratory analyses, see Supplementary Information (Sect. 2.8. Complementary analyses).

## Results

Dominance was significantly associated with Machiavellianism, leadership, hyper-competitive attitude, as well as competitive and critical sibling relationships. DoPL-D was significantly associated with prestige (DoPL-P) (*r* = 0.27, *p* = 0.042), leadership (DoPL-L) (*r* = 0.58, *p* < 0.001), hyper-competitive attitude (HCA) (r = 0.46, p < 0.001), Machiavellianism (Mach-IV) (*r* = 0.51, *p* < 0.001), and Dominant-Leadership subscale (RSPQ) (*r* = 0.37, *p* = 0.005). There were also significant correlations between DoPL-D and negative aspects of the sibling relationship (STQ) such as competitiveness (*r* = 0.48, *p* < 0.001), criticism (*r* = 0.49, *p* < 0.001), and apathy (*r* = 0.36, *p* = 0.006). However, DoPL-D did not show any significant correlations with PDCA (*r* = 0.18, *p* = 0.174), sense of power (PSPS) (*ρ* = 0.24, *p* = 0.074), Coalition-Building (RSPQ) (*r* = − 0.15, *p* = 0.278), or Ruthless Self-Advancement (RSPQ) (*r* = 0.24, *p* = 0.072). For further details, see Supplementary Table S6 and Fig. S4.

### Cluster results

**Figure 3:**
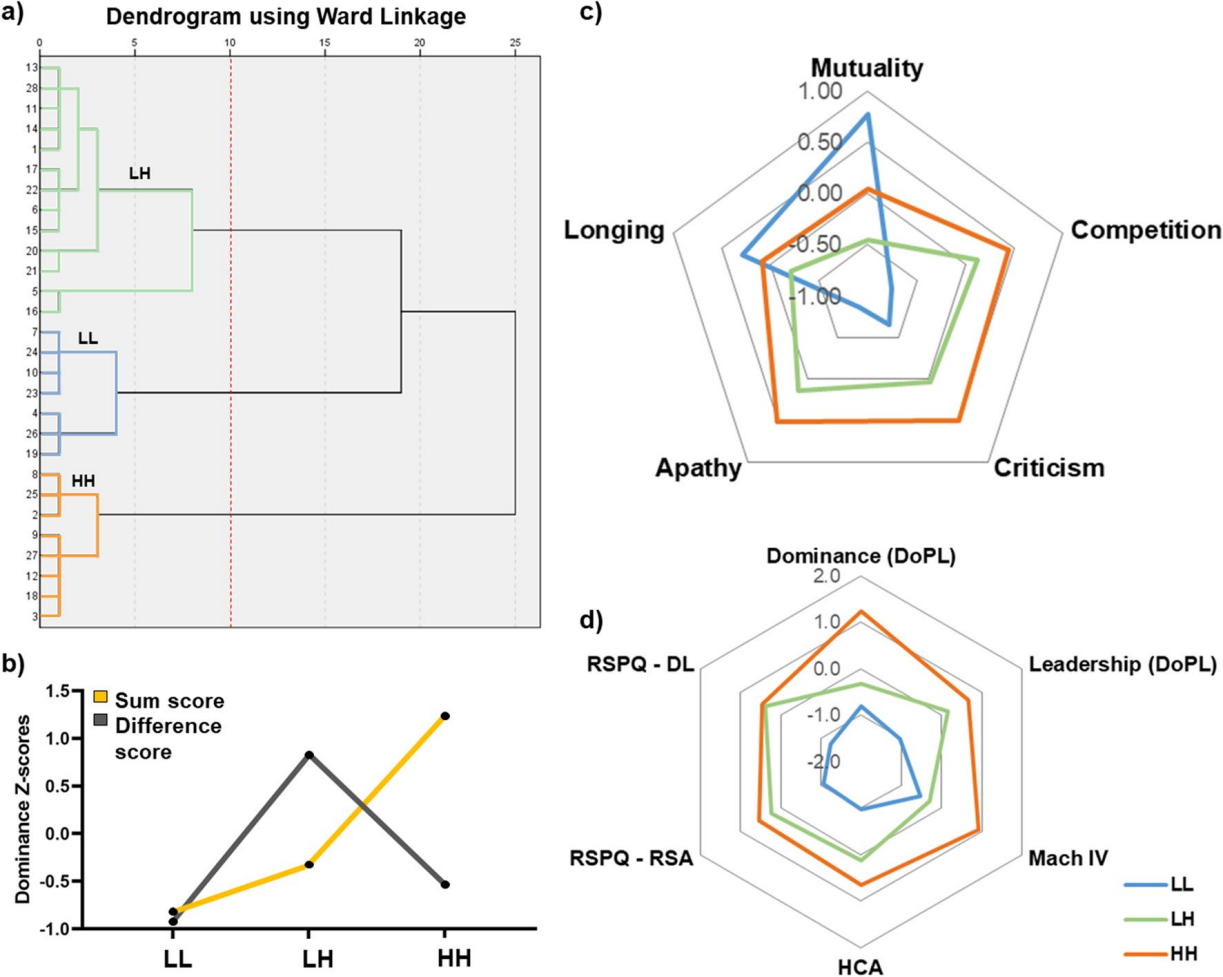
Hierarchical cluster results. (**a**) Dendrogram from hierarchical clustering (Ward’s method, Euclidian distance, SPSS 20.0) showing the sequences of merges and splits of cluster and an optimal solution of 3 clusters, using 10 distance as cutting point (red line). (**b**) The line graph shows the z-standardized mean of the sum of dominance pair scores and the absolute difference between dominance pair scores for each cluster. (**c**) This spider graph represents the z-standardized mean of the Sibling Type Questionnaire (STQ) scores for each cluster. (**d**) This spider graph represents the z-standardized mean of the personality traits scores with significant differences between clusters. *Note*: LL = both low in dominance, LH = one low in dominance, and one in high dominance, HH = both high in dominance, DoPL = Dominance, Prestige, Leadership scale, Mach-IV = Machiavellianism scale, HCA = Hypercompetitive Attitude scale, RSPQ = Rank Style with Peers Questionnaire, DL = Dominant Leadership, RSA = Ruthless Self-Advancement.

The results of cluster analysis based on the sum of pair dominance scores (DoPL-D), and the absolute difference in pair dominance scores indicated three clusters (see Fig. 3 and Table S8). The so-called LL cluster, characterized by both siblings having low dominance scores (*M* = 40, *SD* = 6.27) and the difference between the dominance scores being small (*M* = 2.57, *SD* = 1.72), consisted of 7 pairs. The HH cluster (n = 8 pairs) included both individuals with the highest dominance score (*M* = 58.82, *SD* = 3.43) and the difference between siblings’ dominance scores was also low (*M* = 4.32, *SD* = 2.42). Finally, the LH cluster was formed by 13 pairs and contained one sibling with a lower dominance score and one sibling with a higher dominance score, therefore, the mean of the dominance cluster was among the other clusters (*M* = 44.48, *SD* = 5.89), and the difference between sibling scores was high (*M* = 10.36, *SD* = 3.38).

No significant differences were found in age (*F*(2,25) = 0.666, *p* = 0.523), age difference (*F*(2,25) = 0.129, *p* = 0.879), gender (*X*^2^(2, n = 28) = 4.14, *p* = 0.126), or any other demographic variable, see Table S7 for details.

The comparison of the sibling relationship (STQ) scores between the clusters (Fig. 3c, for statistical results on the STQ, see Table S8), indicated a significant effect of cluster on mutuality. While the HH cluster did not significantly differ from the other clusters, the LL cluster scored higher on mutuality than the LH cluster. Clusters also differed in competition, but post-hoc tests were not significant. For apathy, significant differences were found between clusters and pairwise tests indicated significantly higher apathy in the HH than LL cluster (Table S8). Other STQ scores were not significantly different between clusters (Table S8).

Regarding other self-report measures (for statistical test results see Table S8), there was a significant difference in DoPL-D and DoPL-L between LL, LH, and HH clusters, with LL having lower scores than LH and HH in leadership, while HH showed the highest scores, compared to LL and LH in dominance. Similar results were observed for Machiavellianism scores, with the HH cluster showing significantly higher scores than the other two clusters. The LL cluster showed significantly lower scores on the HCA as well as on the RSPQ (dominant leadership, and ruthless self-advancement subscales) than the other clusters. While the Kruskal–Wallis test indicated significant differences between the clusters for the PSPS, the post-hoc tests were not significant, see Fig. 3d and Table S8 for statistics and further details.

### Cooperative tetris task results

**Figure 4:**
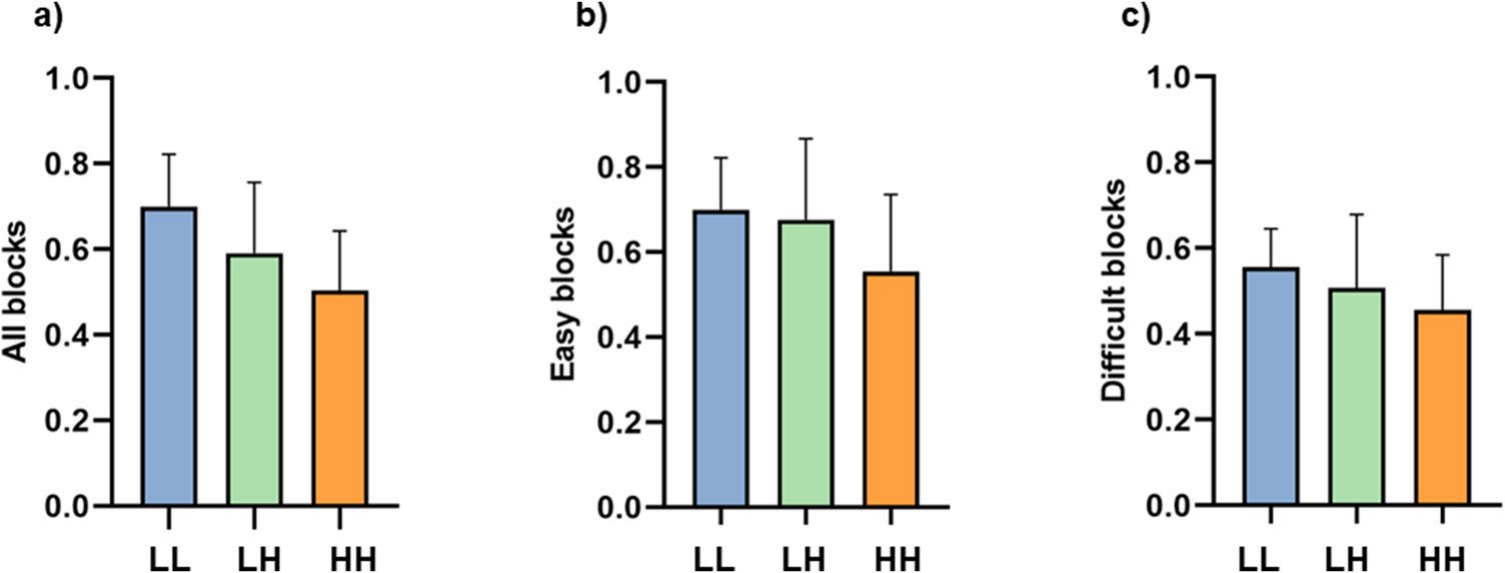
Bar plots representing the mean number of cleared lines (success) per block across the different clusters*.* (**a**) Mean success per block, (**b**) success in easy blocks, (**c**) success in complex blocks. *Note*: LL = both low in dominance, LH = one low in dominance, and one high in dominance, HH = both high in dominance. Error bars represent standard deviation.

There was a significant main effect of block difficulty (*F*(1,25) = 26.19, *p* < 0.001, η_p_^2^ = 0.512) with more cleared lines in easy blocks (*M* = 0.64, *SD* = 0.178) than complex blocks (*M* = 0.51, *SD* = 0.143). There was no significant interaction effect of difficulty and cluster (*F*(2,25) = 0.639, *p* = 0.536, η_p_^2^ = 0.049). Likewise, there were no significant differences between clusters regarding the number of cleared lines per block (*F*(2,25) = 1.50, *p* = 0.240). However, these results revealed a non-significant trend showing the highest number of cleared lines in the LL cluster and the lowest number in the HH cluster (see Fig. 4). For results on the relationship between success and task questions see Supplementary Information (Sect. 3.2).

### Interactive chicken game results

**Figure 5:**
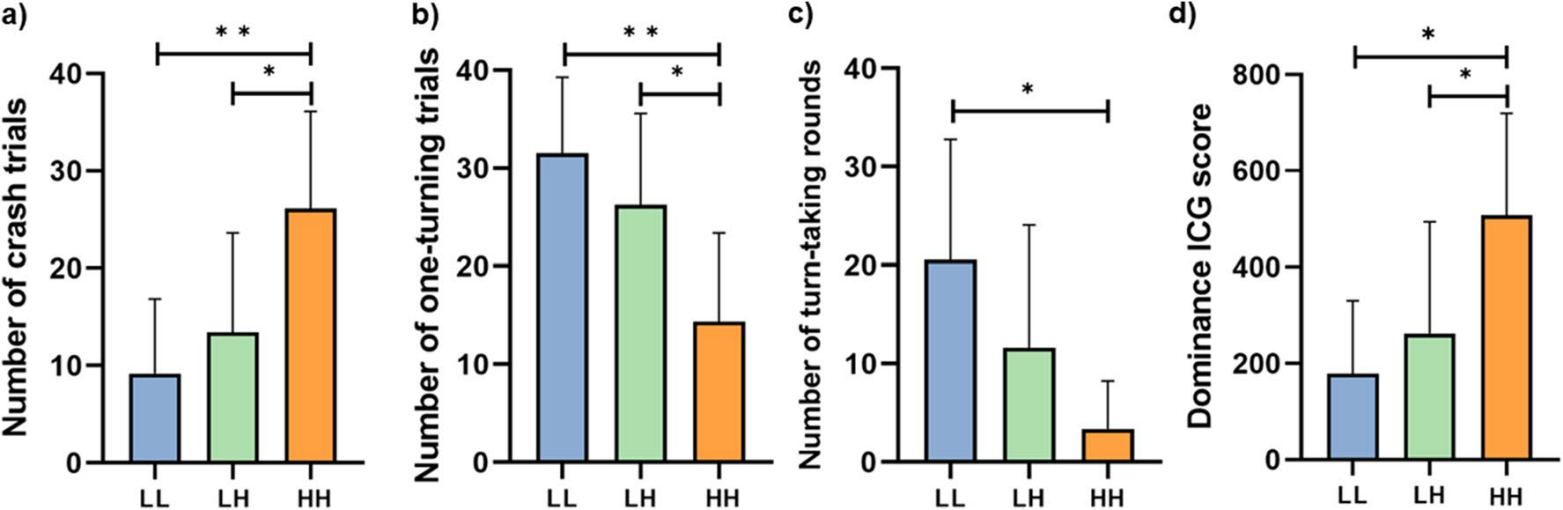
Results of cluster differences in Interactive Chicken Game task across the different conditions: (**a**) mutual defection (DD, dominant behavior), (**b**) one-turning (DT/TD), (**c**) turn-taking rounds (repeat what the other participant did in the last trial, cooperative behavior), (**d**) dominance ICG score. *Note*: LL = both low in dominance, LH = one low in dominance, and one high in dominance, and HH = both high in dominance. Error bars represent standard deviation. Asterisks indicate a significant between-group effect (post-hoc), * p < 0.05, ** p < 0.01. Bonferroni corrected.

Clusters differed in the total ICG score, i.e., sum feedback points, and dominance ICG score, i.e., fewer turns and more crashes (see Table S3 for detailed description).The HH cluster had the lowest total ICG score and highest dominance ICG score (Fig. 5d) while the LL cluster had the highest total ICG score and the lowest dominance ICG score (for statistical results on the ICG see Table S9). The HH cluster also had significantly more crashes than the LH and LL clusters (Fig. 5a). Clusters did not differ in the number of mutual cooperation (both turn). However, there was a significant effect of cluster on the number of one-turning condition with a significantly lower number in the HH cluster compared to the LL and LH cluster (Fig. 5b). The number of turn-taking rounds was significantly higher in the LL cluster than in the HH cluster (Fig. 5c). See Supplementary Fig. S5 for examples of different representative behaviors by cluster (including an example of a turn-taking strategy). There was no significant effect of cluster in comparing the mean differences in total ICG scores. For results on the relationship between dominance and game questions see Supplementary Information (Sect. 3.5).

## Discussion

The current study aimed to validate two novel tasks exploring cooperative and competitive behavior over time in young adult sibling pairs. A second aim targeted the influence of personality traits on sibling’s interactions. Pairs were classified based on the combination of their individual dominance scores (DoPL-D), leading to three different groups (both high in dominance, both low in dominance, and one high and one low in dominance). The group formed by individuals with higher scores in dominance also had higher Machiavellianism, hypercompetitiveness, and apathetic sibling relationships. The associations of dominance and Machiavellianism, hypercompetitiveness attitude, and leadership are mostly in line with previous literature^41,43–45^. High dominant siblings’ interactive behavior was characterized by more car crashes, and less use of one-turning condition and turn-taking strategy than the other two groups supporting that their dominant behavior led to reduced cooperation. In the cooperation task, results showed only a non-significant trend for more success in the cooperative tetris task in low dominance pairs. We will critically interpret the group differences in the tasks, and further discuss how personality and sibling relationships might influence social interactions.

When studying cooperation and competition, which are intrinsically interpersonal activities, a focus on only one person within a dyad is theoretically deficient^32^. We need to characterize behavior in the context of with whom someone is interacting. Cooperation increases after successful interactions^61^, and with partners described as more agreeable and open to experience^33^, but decreases with “partners” denoted with a personality disorders description (e.g. antisocial, avoidant, borderline, or narcissistic)^34^. When it comes to sibling research, the dynamics are unique in terms of the longevity of the relationship^1^, which increases the amount of competitive and cooperative interactions in which the personality is well known to the other. Although previous research has observed that rivalry and conflict are not as prevalent in adult siblings^4^, we have observed that this depends on the personality of both siblings.

Groups differed in their sibling relationship. Sibling pairs formed by two low dominant individuals reported higher mutuality, implying higher affection, acceptance, and reciprocity^55^ than heterogeneous sibling pairs, as well as less apathetic relationship—more interpersonal contact and similarity—than the homogeneously high dominant sibling pairs. This shows that the combination of dominance characteristics is highly related to the sibling relationship. Similar findings have been reported in siblings during adulthood^62^. A close relationship is associated with a higher positive affect and fewer power struggles^16^, and sibling attachment predicts conflict and cooperation^18^. Reports of more sibling conflict in childhood correlated with higher levels of Machiavellianism and psychopathy in adulthood^63^. Importantly, our findings suggest that low dominance motivation not only in one of the siblings but in both siblings supports a good sibling relationship.

Unlike most studies using simulated versions of the CG task or other game theory paradigms^27,30–34^, our Interactive Chicken Game task allowed us to explore natural interactions with a richer game structure. In homogeneously high dominant pairs we observed more car crashes, and lower use of one-turning condition and turn-taking strategy. This led to a higher dominance ICG score during the game for both participants and indicates that these pairs were less cooperative in this dilemma context^49^.

The higher Machiavellian and competitiveness traits in this group may also have contributed to the defective and less cooperative behavior as observed in previous game theory tasks^38,49,64,65^. Similar to our task, the temptation to exploit others predicted cooperation in the CG task^66^. This pattern has also been replicated in children where cooperative dyads used a turn-taking strategy in a modified version of the CG, while among pairs who did not adopt that strategy, the dominant children got higher payoffs^67^. Prior studies have highlighted that more defection in the PD task is linked to greater attempts to maximize one's own gain^68^, which is likely the motivation for individuals with high Machiavellian, competitiveness, and dominance traits to defect. It is important to note that the behavioral differences were not due to group differences in demographic variables (sex, age, age difference, or socio-economic status).

Low dominant pairs used a turn-taking strategy to maximize their gains, and let the other win as many points. Supposedly, they expected the same cooperative treatment from the other^69,70^, which would be in line with the good relationship the low dominant siblings indicated. Interestingly, both subordinate and heterogeneous pairs showed a similar pattern of behavior in the task, which may indicate that one person who has low dominance is sufficient for a better outcome in social dilemma tasks.

In contrast to other puzzle-solving tasks, the cooperative tetris task combined both real interaction and a semi-controlled experimental design that enabled equal opportunities at the start of each block and the manipulation of trial complexity. While we could show that the manipulation of complexity worked, the performance of sibling pairs was similar independent of their dominance structure. We found little support that siblings who were both low in dominance—and more cooperative—would perform better, although the descriptive evidence points in the predicted direction.

Previous literature showed that Machiavellianism and dominance seem to be negatively associated with team performance and team cooperation^46,47,71^. Teams formed by largely dominant or leader-type people significantly performed worse on a collaborative task^46^. Having a group member with high dark personality traits has been associated with a toxic and detrimental relationship with more subordinate members^72–75^ which has a negative impact on performance. Since these dark personality traits were different in our pairwise sibling groups, the missing difference in team performance during puzzle solving either indicates that cooperation in our tasks was not dependent on these characteristics or that other factors had a stronger influence. In addition, siblings may have a stronger cooperative basis than other persons^76^, which may reduce the influence of characteristics that usually inhibit cooperative behavior between unfamiliar persons. Indeed, all teams were highly motivated to play as a team and siblings’ willingness to play as a team was not related to their success. Thus the engaging nature of the task, also supported by the consistent participants’ ratings of the task being joyful, may have reduced the influence of problematic characteristics or relationships.

These results support the reciprocal influence between the individual's personality and the sibling relationship. However, it is not possible to know the causal direction of whether more dominant individuals cause a more apathetic sibling relationship, or whether it is the less affective and close relationship that causes higher dominant and Machiavellian traits. Our interpretation is that this is probably a circular relationship in which the sibling’s personalities and the relationship influence each other. This highlights the importance of sibling interaction even in adulthood due to its influence on people’s personalities and relationships. Another possible explanation could be that there is no direct causal relationship between dominance and sibling relationship, but that the correlation may exist due to some unknown factor causing both, such as parental influences, e.g. attachment styles.

The main advantage of our tasks is the level of interaction and the engagement setting that allows us to study social interactions in real-time and over time. One of the most important shortcomings of game theory tasks is their limited ecological validity related to their oversimplified binary response options, which restrict the analyses of more complicated interactions like competition and trustworthiness^25^. The Interactive Chicken Game task addresses this limitation by incorporating real-time interaction and offering the possibility of turning at any time for 5 s. Participants not only had the binary choice of turning or going straight, but had to adjust their choices to the changing gain and loss options. The video with the driver’s first-person perspective approaching the other car increases immersion and realism compared to the standard setup were participants only choose between the two options. In the cooperative tetris task, although participants adopt different roles, they interact together, rather than taking turns as in other puzzle-solving tasks^25,27^. In both tasks, the two participants are in the same decision phase with the same changing decision context, which provides an advantage for electrophysiological and neuroimaging studies, performing for example hyperscanning. This novel technique allows the simultaneous recording of brain activity measuring interbrain synchronicity^77^. Our tasks do not require face-to-face interaction. Therefore, they are an excellent tool to study social interaction in real-time, even in functional magnetic resonance imaging hyperscanning environments.

## Limitations

The present study included 56 participants, but as most of the analyses were conducted pairwise, the sample size was 28 pairs, divided into 3 groups. This strongly limits the ability to detect significant group effects and may reduce the robustness of these effects. The small sample size could be the reason for the lack of significant differences in the cooperative tetris task. We computed a post-hoc analysis using G*Power (version 3.1.9.7.), and indicating a sample size of 190 pairs would be needed to find a significant effect with a power of 0.8.

Although we created a richer game structure than standard experimental games, the tasks may not reflect real-life situations. Both paradigms are closely associated with computer games, but the extrapolation to day-to-day cooperation or competition remains limited and should be improved in future studies.

Finally, we only included same-sex sibling pairs due to the limited time and funding of this study consequently limiting the generalization of our results. The results on sex differences (brother vs sister pairs) and their interpretation can be found in the Supplementary Information (Sect. 3.6) but must be considered with caution. Future studies should examine the differences between same-sex and opposite-sex siblings in competitive and cooperative tasks in larger samples.

## Conclusion

Beyond previous observational studies, the current results demonstrate different patterns of sibling relationships in emerging adulthood associated with both siblings’ personalities. Pairs consisting of two dominant, Machiavellian, and hypercompetitive individuals had a more apathetic sibling relationship and showed more dominant behavior through higher defection (Interactive Chicken Game task). Instead, pairs consisting of two subordinate and cooperative individuals showed the opposite pattern by taking turns to maximize their gains and having more support and affection. Heterogeneous pairs showed intermediate performance. None of the groups significantly differed in puzzle solving performance (cooperation tetris task). These group differences underline the importance of studying social interactions in siblings’ young adult life concerning their impact on personality and relationships. Differences in cooperative and competitive behavior are found in real-time interactions over time by observing the natural patterns that arise from real social exchange between related individuals. These tasks increase the interactivity and engagement of participants, providing more realistic and ecologically validated measures compared to standard experimental tasks.

## Supplementary Information

Supplementary Information.

## Data Availability

Supplementary data to this article can be found online. The Cooperative Tetris Task and Interactive Chicken Game tasks (Psychopy) as well as the datasets generated and/or analyzed during the current study are available from the corresponding author upon request. The questions included in the tasks are available in the Supplementary Information file. The Tetris blocks’ design and graphs showing the behavior of each pair during the Interactive Chicken Game are available in the OSF pre-registration of the project (https://osf.io/ch6ug/).
