# Supplementary material for: The role of dominance in sibling relationships: differences in interactive cooperative and competitive behavior
Source: Sci Rep. 2023 Jul 22;13:11863. doi: 10.1038/s41598-023-38936-7 (PMC10363155; doi:10.1038/s41598-023-38936-7)
Supplement: Supplementary file 1 — Supplementary Information. [file 41598_2023_38936_MOESM1_ESM.pdf]

Supplementary material: The role of dominance in sibling relationships: differences in interactive cooperative and competitive behavior

Lucia Hernandez-Pena[1,2*], Wiebke Hoppe[1], Julia Koch[1,2], Charlotte Keeler[1], Rebecca Waller[3], Ute Habel[1,4], Rik Sijben[5], Lisa Wagels[1,2]

[1] Department of Psychiatry, Psychotherapy and Psychosomatics, Faculty of Medicine, RWTH Aachen, Pauwelsstrasse 30, 52074 Aachen, Germany.

[2] JARA – Translational Brain Medicine, Aachen, Germany.

[3] Department of Psychology, University of Pennsylvania, Philadelphia, PA, USA.

[4] Institute of Neuroscience and Medicine: JARA-Institute Brain Structure Function Relationship (INM 10), Research Center Jülich, Jülich, Germany.

[5] Brain Imaging Facility, Interdisciplinary Center for Clinical Research (IZKF), RWTH Aachen University, Aachen, Germany.

* Corresponding author: Lucia Hernandez-Pena

Department of Psychiatry, Psychotherapy and Psychomatics, Uniklinik RWTH Aachen, Germany; Pauwelsstrasse 30, 52074 Aachen, Germany; E-mail: lhernandez@ukaachen.de; Phone: +49 241 80-85333.

1. Supplementary Introduction 4
1.1. Extended hypotheses 4
2. Supplementary Methods 5
2.2. Programs and Set-up 5
2.3. Creation of the Tetris Blocks 5
2.3.1. General Criteria 5
2.4. Evaluation of the Tetris Blocks 8
2.4.1. Materials and Procedure 8
2.4.2. Evaluation Analyses 9
2.4.3. Results comparing the final blocks for the Cooperative Tetris Task task 10
2.5. Measures description 12
Supplementary Table S3. Description of Measures in the Tasks. 12
2.6. Task questions 13
2.6.1. Cooperative Tetris Task questions 13
Supplementary Table S4. 13
2.6.2. Interactive Chicken Game questions 14
Supplementary Table S5. 14
2.7. Questionnaires 16
2.8. Complementary analyses 17
2.8.1. Cooperative Tetris Task analyses 17
2.8.2. Interactive Chicken Game analyses 17
3. Supplementary Results 19
3.1. Questionnaires Correlations 19
Supplementary Table S6. Correlations between Questionnaire Scores 19
Figure S4. 20
Supplementary Table S7. 21
Supplementary Table S8. 23
3.2. Cooperative Tetris Task Correlations 25
3.3. Interactive Chicken Game pair representative behaviors 25
Figure S5. 26
3.4. Interactive Chicken Game performance 26
Supplementary Table S9. 26
3.5. Interactive Chicken Game Correlations 26
3.6. Sex Differences 27

Supplementary Table S10. T-test results comparing brother and sister pairs on various measures. 27

References 28

1. Supplementary Introduction

1.1. Extended hypotheses

We expected individual and group differences according to these hypotheses:

a. Dominant individuals (high scores on the Dominance subscale of the DoPL; DoPL-D) would show higher scores on the Criticism, Competition, and Apathy subscales of the Sibling Type Questionnaire (STQ), as well as higher scores on the Prestige and Leadership subscales (DoPL-P, DoPL-L), the Personal Development Competitive Attitude Scale, the Hypercompetitive Attitude Scale, Machiavellianism scale (Mach – IV), the Personal Sense of Power Scale and on Dominant Leadership and Ruthless Self-Advancement factors in the Rank Style with Peers Questionnaire.

b. We expected to find personality-paired groups (subordinate–subordinate, subordinate–dominant and dominant–dominant pairs) that would differ in terms of their sibling relationship characteristics, as well as in their scores on questionnaires measuring dominance, competitiveness, and Machiavellianism; with the dominant–dominant group showing a more highly competitive, critical, and apathetic sibling relationship, as well as higher scores on the questionnaire measures.

In the *Cooperative Tetris Task* (CoTT), we expected:

c. Performance would be better in easy blocks than in complex blocks.

d. Subordinate–subordinate pairs would perform better than subordinate–dominant pairs, and dominant–dominant pairs.

e. Positive feedback between players would be positively associated with performance.

f. The sibling who acts (movement or rotation) first more frequently would evaluate him/-herself as the leader in the task question and would show higher DoPL-L and DoPL-D.

g. Success would be positively associated with perception of success, shared mental representation, and with willingness to play as a team.

In the *Interactive Chicken Game* (ICG), we expected:

h. Groups would differ in dominance behavior, where 1) subordinate–subordinate pairs would a) have fewer crashes (considered as dominant behavior), and use the turn-taking strategy more frequently (considered as cooperative behavior), and b) have a smaller difference in their total ICG score (similar feedbacks); 2) dominate–subordinate pairs would have a) more crashes than the subordinate-subordinate pairs and fewer crashes than the dominant-dominant pairs and b) a larger difference in their total score (different feedbacks); 3) more

competitive (dominant–dominant) pairs would a) have more crashes and use the turn-taking strategy less frequently, and b) have a smaller difference in their total ICG score (similar feedbacks).

i. Dominant individuals would show higher motivation to win and report the use of more dominant and aggressive strategies during the game, while subordinate/cooperative individuals will report the use of fairness and turn-taking strategies.

j. Brothers would show more dominant behavior (more crashes) than sisters, who will show more cooperative behavior (turn-taking strategy).

2. Supplementary Methods

2.2. Programs and Set-up

A virtual server handled any communication for the experimental paradigm platforms using the socket module in Python 3.6.6. All tasks were synchronized by having the paradigm-presenting computers located at the respective rooms (hereafter referred to as "clients") communicating with this server over TCP/IP. These clients sent paradigm-relevant information like "readiness" and trial information to the server. Upon receiving specific messages from both clients, the server replied to both clients with an appropriate message. When both clients were connected to the server, the experimenter manually started the Cooperative Tetris Task for both clients at the same time, sending a message to Psychopy through the server. For the Interactive Chicken Game, once both clients were connected to the server, the latter automatically started the task. In both tasks, the server waited for the confirmation of each client to ensure that both computers were synchronized with a maximum latency of approximately 16 milliseconds at the beginning of each trial.

2.3. Creation of the Tetris Blocks

2.3.1. General Criteria

A set of criteria was determined before creating the new Tetris blocks intended for use in the CoTT. Tetris blocks were structured as follows:

1. Each block would start with uniquely preassembled base pieces.
2. In every *block*, four Tetris pieces would need to be placed among the base pieces. Each Tetris piece represents one *trial*.
3. Two types of blocks were defined:
    a) Easy blocks (Fig. S1):

- Every Tetris piece placed must be able to complete a line if the correct position and rotation are chosen (optimal position).
- Every Tetris piece placed must have one unambiguous optimal position to fit into and only choosing this option completes a line.

b) Complex blocks (Fig. S2):

The complex blocks are formed by two different type of trials:

- Two-option (complex) trials: The first trial must have exactly two unambiguous equally optimal positions to fit into but that does not delete a line. Optimal positions are defined as empty spaces in which if a piece is placed that fits in that spot, there is only one space left for the next piece to complete the line. If the players use the pieces as planned, the third trial again offers two equally optimal positions by which no line can be completely filled.
- One-option (easy) trials: The second and the fourth Tetris piece must have the potential to complete a line (given that the optimal position was used in the trial before). They must have an unambiguous optimal position to fit into and there can only be one option that can complete a line.

Note, that players could still place the Tetris pieces in positions not further described here although we tried to make it as obvious as possible to the players which position(s) is optimal for success. A piece that is positioned less optimal and/or the following pieces might not have the potential to clear lines as intended.

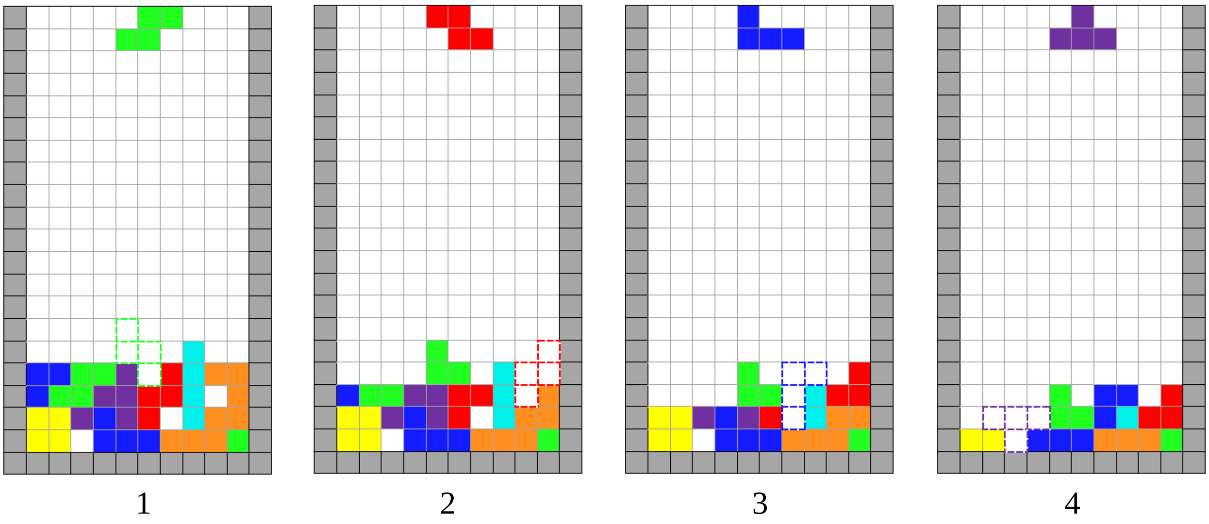

Figure S1. Easy Block with four One-Option Trials**.** This figure shows the four One-option trials of an easy block, and a representation of the optimal solution to them. The dashed lines indicate the ideal position for the piece that descends from the top in order to complete and clear a line.

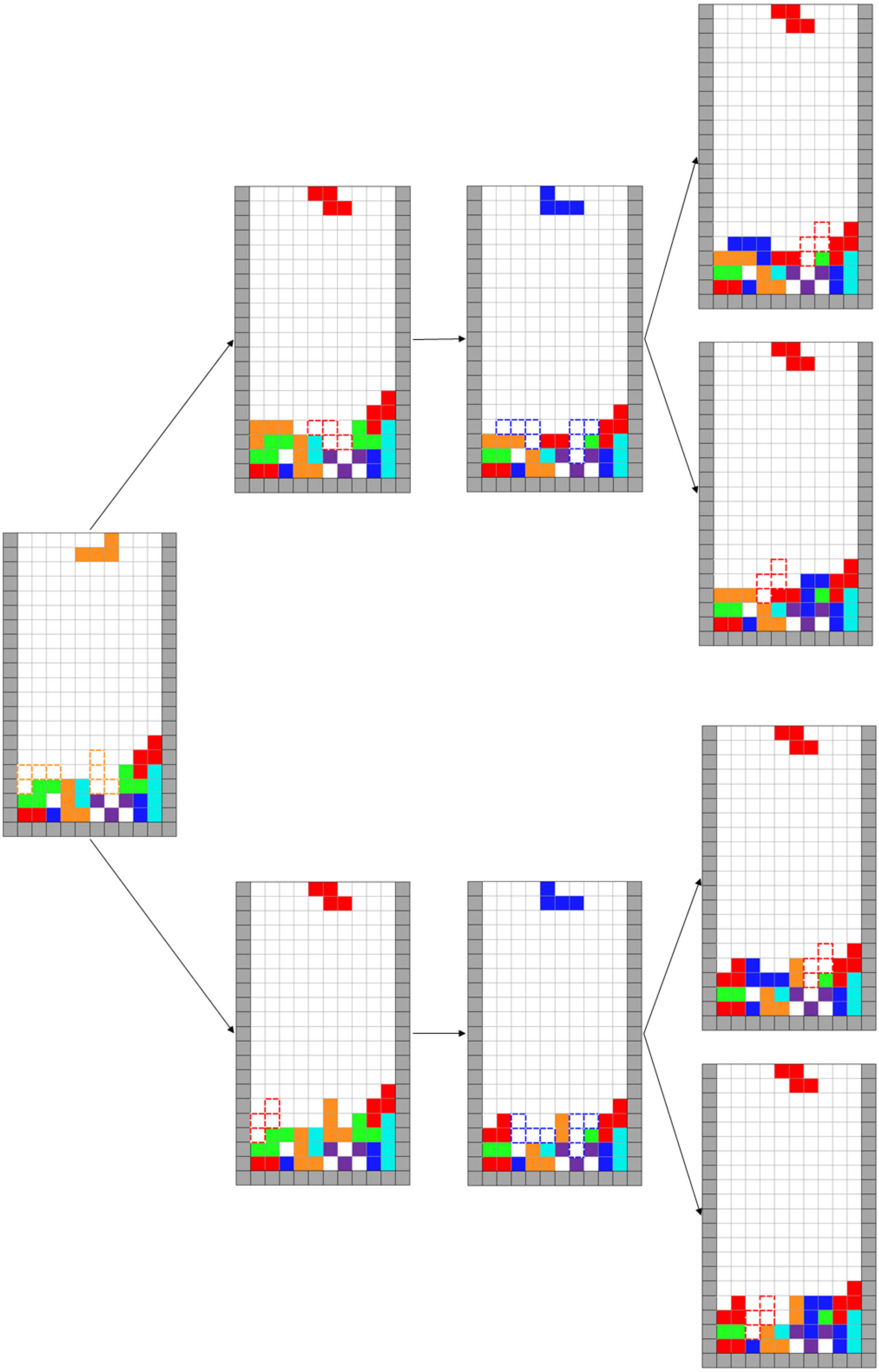

1 2 3 4

Figure S2. Complex Block with two Two-Option Trials and two One-Option Trials.

This figure shows an example of the four trials of a complex block and a representation of the optimal solutions to them. The dashed lines indicate the ideal position for the Tetris piece to complete and clear a line within this or the following trial. Odd trials have two ideal positions (Two-option trials) and even trials one (One-option trials). In each Two-option trial the chosen ideal location of the piece determines the ideal location of the next piece, thus resulting in two trial variants for the following One-option trial (depicted by split arrows).

2.4. Evaluation of the Tetris Blocks

2.4.1. Materials and Procedure

We created a total of 22 easy blocks and 22 complex blocks. We conducted a pilot study in which we presented the trials of these 44 blocks to 16 independent participants (8 males, 8 females, age range from 20 to 35 years) and asked them to indicate their first, second, and third choice of positions (later described as "field preference") in which they would place the predefined Tetris piece. For each of these three choices, they were asked to rate the likelihood of choosing this position. They were also asked whether they think that there are more than three possible positions for the piece. Additionally, participants evaluated the level of difficulty of the block (Fig. S3).

Each easy block and each complex block were composed of four trials. This made a total of 88 easy trials across all easy blocks. Since complex blocks are presented in different variations (Fig. S2) the complex blocks contained easy (one optimal option) and complex (two optimal options) trials. Thus, there were three Two-option trials (trial 1, trial 3A, trial 3B) and six One-option trials (trial 2A, trial 2B, trial 4A, trial 4B, trial 4C, trial 4D), adding up to a total of 198 to-be-evaluated trials. Four of these trials (two Two-option trials, and two One-option trials) were deleted because we retrospectively realized that they were duplicates of other trials in the same block and seven of the complex blocks were an inverted version of another complex block. Summing up, a total of 65 complex trials and 217 easy trials for a total of 44 blocks were evaluated in the pilot study.

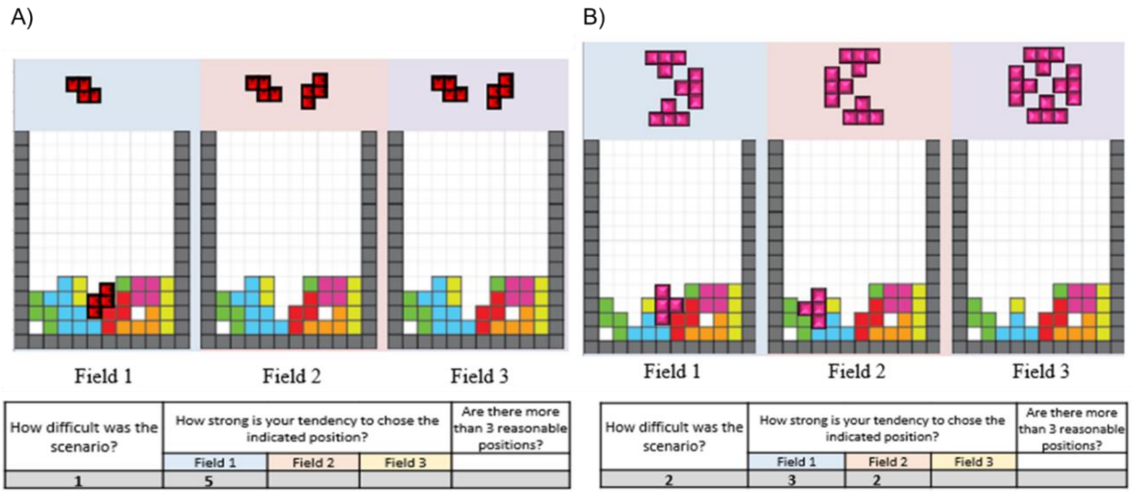

Figure S3. Example of an Evaluation of A) One-Option (easy) Trials and B) Two-Option (complex) Trials.

Participants received an Excel file with all the trials to evaluate. We alternated the easy and complex blocks so that all evaluations started with four easy blocks and were followed by four complex blocks etc., in the same order for all participants.

2.4.2. Evaluation Analyses

We compared the difficulty between easy and complex blocks and the tendency for one or two fields. The difference in difficulty between easy and complex blocks was significant, $F(1,14) = 51.28$, $p < 0.001$, with easy blocks, indeed evaluated as easier (M = 1.65, SD = 0.15) compared to complex blocks (M = 1.85, SD = 0.16). The interaction between difficulty and field tendency was significant as well, $F(1,14) = 6.65$, $p = 0.022$, showing that people generally indicated only one solution more strongly (M = 4.82, SD = 0.059), compared to the use of any other field (M = 2.82, SD = 0.072) in easy blocks.
Further criteria to select blocks were the mean difficulty and strength of preferences as well as the number of orientations per piece which should be as equal as possible for all blocks. We accepted a combination of two simple pieces (2 possible orientations, 1 rotation) and two complex pieces (4 possible orientations, 3 rotations) or one simple piece and three complex pieces; note that pieces could only be turned in one direction.

Selection Easy Blocks. Those blocks with a lower mean difficulty were selected as easy blocks. In six of the selected blocks, all pieces could be optimally placed by at least 12 possible piece orientations (2 simple and 2 complex pieces) and the other six by at least 14 possible piece orientations (1 simple and 3 complex pieces).

Selection Complex Blocks. The final 12 complex blocks were selected by the highest scores in difficulty and by the tendency for field options. The following assumptions about the Two-option trials guided our analyses:

1) The difference in the strength of participants' tendency towards the position indicated in field 1 (first position option) and their tendency towards the position indicated in field 2 (second position option) was expected to be small because the Two-option trials were designed in such a way that they would suggest two equally advantageous positions for achieving success (filling and clearing a line).
2) The difference in the strength of participants' tendency towards the position indicated in field 2 and their tendency towards the position indicated in field 3 was expected to be greater than the difference between field 1 and field 2 because the Two-option trials were designed in such a way that they would suggest only two advantageous positions for achieving success (filling and clearing a line) and not more.

Based on these considerations, we selected the 12 complex blocks calculating complex trials within complex blocks (the three Two-Option trials) that have the lowest difference between fields 1 and 2 and the greatest difference between fields 2 and 3. To take both criteria into account at the same time, we subtracted the difference between fields 1 and 2 from the difference between fields 2 and 3 and chose the blocks with a lower difference (meaning that they have a small difference between 2 and 3 and a great difference between 1 and 2).

Using a one-way ANOVA, we again compared the mean difficulty between the easy and complex blocks of the finally selected blocks. We also tested if there was a significant difference between the first preferred and the second preferred option in the complex trials, assuming that there would be no significant difference.

2.4.3. Results comparing the final blocks for the Cooperative Tetris Task task

The difference in difficulty between easy (M = 1.57, SD = 0.56) and complex (M = 1.88, SD = 0.63) blocks was significant, $F(1,14) = 90.20$, $p < 0.001$.

The difference in the tendency towards the first and second preferred field in easy and complex blocks was significant as well, $F(1,14) = 14.68$, $p = 0.002$, $\eta_p^2 = 0.51$ for further details see supplementary Table S1.

Supplementary Table S1. ANOVA Main and Interaction Effects

	F(1,14)	P	η_p^2
Difficulty	90.20	**< 0.001**	0.87
Difficulty * Sex	1.29	0.276	0.08
Field tendency	1.37	0.261	0.09
Field tendency * Sex	1.29	0.273	0.09
Difficulty * Field tendency	14.68	**0.002**	0.51
Difficulty * Field tendency * Sex	9.02	**0.010**	0.39

Note. Statistically significant values are shown in bold.

Post-hoc tests showed that the tendency towards field 1 was significantly different from the tendency towards field 2 in easy blocks, $F(1,14) = 26.36$, $p < 0.001$, $\eta_p^2 = 0.65$, while no significant differences were found comparing the tendency for field 1 and field 2 in complex blocks, $F(1,14) = 0.94$, $p = 0.349$. $\eta_p^2 = 0.06$, demonstrating that both options were preferred equally (for further details see supplementary Table S2).

Supplementary Table S2. Post-hoc Tests of Difficulty and Field Tendency Interactions

				Post-hoc statistics		
Post-hoc contrast		M	SD	F(1,14)	P	η_p^2
Easy	Field 1	4.86	0.05	26.36	**<0 .001**	0.65
	Field 2	4.65	0.07			
Complex	Field 1	2.88	0.21	0.94	0.349	0.06
	Field 2	2.97	0.18			
Field 1	Easy	4.86	0.05	84.48	**< 0.001**	0.86
	Complex	4.65	0.07			
Field 2	Easy	2.88	0.21	91.22	**< 0.001**	0.87
	Complex	2.97	0.18			

Note. Field refers to the preference for the first and secondary choice. Statistically significant values are shown in bold.

Interactions between difficulty and gender, as well as field tendency and gender, were not significant.

2.5. Measures description

During the tasks, several outcome measures assessing performance and interaction were collected (see Table S3).

Supplementary Table S3. Description of Measures in the Tasks.

Measure	Definition	Formula
**Cooperative Tetris Task**		
**Success easy block**	Mean number of lines cleared in an easy block.	**Mean(easy)** = Σ(number of lines)/48
**Success complex block**	Mean number of lines cleared in a complex block.	**Mean(complex)** = Σ(number of lines)/24
**Total success**	Total number of cleared lines.	**Total success** = Σ(number of lines)
**Initiator of action**	Mean number of actions started by player A/B in easy and complex trials	Initiator (easy) = n(A)/96 and n(B)/96<br>Initiator (difficult) = n(A)/96 and n(B)/96<br>with<br>n(A) = n(RT_A >RT_B)<br>n(B) =192 - n(A)
**Interactive Chicken Game**		
**Mutual defection – both crash (DD)**	Both crash: trial in which none of the participants decided to turn.	**DD** = Σ(crashes)
**Mutual cooperation – both turn (TT)**	Both turn: trial in which both participants turned in the same interval, losing the same number of points.	**TT** = Σ(both turning)
**One-turning (TD, DT)**	One dominant, one subordinate: trial in which one of the participants turned and the other participant wins the trial.	**TD/DT** = Σ(A turns, B wins) + Σ(B turns, A wins)
**Turn-taking strategy**	Number of trials in which the participant does the same thing as the other participant did in the previous trial (not counting both turning, and both crashing). E.g., participant A turns in second 1, and participant B turns in the next trial in second 1. See Fig. S5 for a turn-taking strategy example.	**Turn-taking strategy** = [Σ(decision response A == previous decision response B) + Σ(decision response A == previous decision response B)] – DD – TT
**Total ICG score**	Sum of the points received by the participants throughout the task. Higher total ICG scores mean less dominant behavior across the task (fewer negative feedback points). Crashing corresponds to -10 points.	**Total ICG score** = Σ(feedbackA + feedbackB)| with crashes = - 10 points

**Difference total ICG score**	Difference of the final score of both participants within a pair, in which crashing corresponds to -10 points.	**Difference total ICG score** =|Final score A - final score B| with crashes = - 10 points
**Dominance ICG score**	Sum of the dominant behavior of the participant during the task, in which crashing corresponds to 10 points, winning because the other has turned corresponds to points gained, and turning subtracts points lost. Higher dominance ICG score means more dominant behavior across the task.	**Dominance ICG score** = Σ(feedbackA + feedbackB)| with crashes = +10 points

Note. ICG = Interactive Chicken Game, RT = Response time.

2.6. Task questions

2.6.1. Cooperative Tetris Task questions

After each round of the Cooperative Tetris Task, participants provided feedback (see Table S4) to their sibling by selecting between positive, neutral, or negative sentences and displayed on the sibling's screen after selection. Further, questions concerning perception of personal success, responsibility for the performance, shared mental representation with their sibling, leadership, and teamwork preference were asked. At the end of the task, participants answered questions about the likability and difficulty of the task, and their prior experience with the Tetris game. For further description of the questions, see Table S4.

Supplementary Table S4. Cooperative Tetris Task Additional Questions Presented in the Task.

Time point	Variable	Question	Response options
Between each of the blocks	Success perception	How successfully were you in the last block?	continuous:<br>0 = Very unsuccessful<br>100 = Very successful
	Outcome responsibility	Which of you is more responsible for the outcome?	1 = Me (100%)<br>2 = Me (75%)<br>3 = Both equally (50%)<br>4 = My brother/sister (75%)<br>5 = My brother/sister (100%)
	Shared mental representation (R)	How similar do you think your joint mental planning was in the last block?<br>-Reversed item-	1 = Very similar<br>2 = Rather similar<br>3 = I don't know<br>4 = Rather different<br>5 = Very different
	Leadership	Which of you had the leading role in the last block?	1 = Me (100%)<br>2 = Me (75%)<br>3 = Both equally (50%)<br>4 = My brother/sister (75%)<br>5 = My brother/sister (100%)
	Team	Would you rather continue playing alone or in a team?	continuous:<br>0 = Alone<br>100 = Team
	Feedback between players (R)	What do you want to say to your brother/sister?<br>-Reversed item-	1 = "You did well!" (positive)<br>2 = "Next round" (neutral)<br>3 = "You blew it!" (negative)

End of the task	Enjoy	How much did you enjoy the game?	1 = Not at all<br>…<br>5 = Extremely well
	Difficulty	How difficult was the game for you?	1 = Not at all<br>…<br>5 = Extremely difficult
	Experience	Have you ever played the game Tetris before?	1 = Never<br>…<br>5 = Very often

Note. These questions/items and response options were originally created for the task. A German version of these questions were used in our study. R = reversed item.

2.6.2. Interactive Chicken Game questions

After each block of the Interactive Chicken Game task, participants answered questions about perceived responsibility for the outcome, motivation, and thoughts and feelings about winning and losing. Feedback was displayed after the block indicating if the player won, lost, or if there was a tie (as a result of the comparison between the sum of the points that each participant gained or lost throughout the trials in the block). At the end of the task, questions about their own and their sibling's expected intentions and strategies during the game were posed. For further description of the questions see Table S5.

Supplementary Table S5. Interactive Chicken Game Additional Questions Presented in the Task.

**Time point**	**Variable**	**Question/Item**	**Response options**
Between each block	Outcome responsibility	Which of you was more responsible for the outcome?	1 = Me (100%)<br>2 = Me (75%)<br>3 = Both equally (50%)<br>4 = My brother/sister (75%)<br>5 = My brother/sister (100%)
	Motivation	In the last block, I had the following thoughts:	0 = "I didn't care who won."<br>…<br>100 = "I wanted to win."
	Win feelings	Please rate HOW YOU FELT after you WON a trial:<br>Use the following scale:<br>I felt powerful.<br>I felt superior.<br>I was afraid of revenge. (reversed)<br>I felt happy.<br>I felt guilty. (reversed)<br>I had the feeling of being dominant.<br>I had the feeling of being fair.	1 = Not at all<br>2 = A little<br>3 = Neutral<br>4 = Considerably<br>5 = Extremely
	Lose feelings	Please rate HOW YOU FELT after you LOST a trial:<br>Use the following scale:<br>I felt like<br>I felt like a failure.	1 = Not at all<br>2 = A little<br>3 = Neutral<br>4 = Considerably<br>5 = Extremely

		I felt inferior.<br>I felt angry.<br>I felt vengeful.<br>I felt happy for my brother/sister. (reversed)<br>I felt sad.<br>I felt it served me right. (reversed)	
End of the task	Statements	To what extent do you think the following statements describe the game so far?	1 = A little<br>…<br>5 = A lot
	Sibling dominant	My brother/sister constantly tries to be in charge of the game.	
	Sibling aggressive	My brother/sister tries to harm me by not swerving, even if it leads to an accident.	
	Participant subordinate	I do not dare to drive on.	
	Perception of fairness	If he/she treats me fairly, I'll be fair too!	
	Sibling subordinate	My brother/sister does not dare to drive through.	
	Perception of coordination	My brother/sister and I try to coordinate to make similar gains.	
	Participant aggressive	I just want to harm my brother/sister.	
	Participant dominant	I'm dominating the game.	
	Perception of turn-taking	I adapt to the strategy of my brother/sister.	
	Strategy	Which strategy did you use most of the time?	single choice:<br>• I repeated what my brother/sister did in the last round.<br>• If I won, I did the same thing again, otherwise I changed my strategy.<br>• I tried to help my brother/sister no matter how he/she acted.<br>• I tried to harm my brother/sister no matter how he/she acted.<br>• I tried to be the superior one, regardless of my brother/sister's behavior.<br>• I tried to play as fair as possible.

Note. These questions/items and response options were originally created for the task. A German version of these questions were used in our study.

2.7. Questionnaires

Siblings were asked to answer questions on their sibling relationship (Sibling type questionnaire, STQ; Stewart et al. 2001). The German translation of the STQ assessed *Mutuality* (12 items), *Competition* (7 items), *Criticism* (13 items), *Apathy* (12 items), and *Longing* (6 items) by 50 statements that could be answered on a 5-point Likert-like scale. Subscale scores were calculated as the sum of the corresponding items. In our sample, the five subscales of the STQ showed good internal consistency (α = 0.92, 0.76, 0.88, 0.65, and 0.49, respectively).

Participants were also asked to answer questions on their personality traits, specifically, dominance behavior was assessed using the German version of the *Dominance, Prestige, and Leadership Scale* (DoPL) [45]. Each subscale (Dominance; DoPL-D, Prestige; DoPL-P, Leadership; DoPL-L) contains 10 items, showing a good internal consistency (α = 0.80, 0.84, and 0.87, respectively), and is answered using a 6-point Likert scale. The total scores for each subscale were calculated using the mean of the corresponding items. In order to measure competitiveness traits, German translations of the *Personal Development Competitive Attitude Scale* (PDCA)[61] and *Hypercompetitive Attitude Scale* (HCA)[43] were assessed. PDCA scale measures competitive attitudes based on personal development goals using 15 items rated on a 5-point Likert scale, and showing good internal consistency (α = 0.85), while the HCA measures general hypercompetitive attitudes, by responding to 26 items on a 5-point Likert scale and showing a questionable internal consistency (α = 0.61). In order to assess Machiavellianism traits, a German translation of the *Machiavellianism Scale* (Mach-IV)[62] was administered. The original version of this questionnaire includes 20 items, but here, two items had to be excluded due to technical problems with the questionnaire administration platform. In total, 16 responses from 6 items were arbitrarily missing. These values were imputed with the mean value. The remaining 18 items were rated on a 5-point Likert scale, showing an acceptable internal consistency (α = 0.72). Scores were calculated as the sum of the questionnaires' items.

To explore their relationship with others, a German translation of the *Rank Style With Peers Questionnaire* (RSPQ)[63] was assessed to measure individuals' social rank style. The subscales *Dominant Leadership* (DL), *Coalition Building* (CB), and *Ruthless Self-Advancement* (RSA) were rated on a 5-point Likert scale and showed good to acceptable

internal consistency (α = 0.86, 0.75, and 0.74, respectively). Also, a German translation of the generalized version of the *Personal Sense of Power Scale* (PSPS)[64] was assessed using a 6-point Likert scale (unintentionally omitting the neutral option) which showed good internal consistency (α = 0.82).

A number of other questionnaires and inventories, including the Buss-Perry Aggression Questionnaire[65], Adult Sibling Relationship Questionnaire[66], Barratt Impulsiveness Scale[67], State-Trait Anger Expression Inventory[68], and NEO Five-Factor Inventory[69] were also part of the survey for other purposes, but are not further considered here.

2.8. Complementary analyses

As an exploratory analysis, we compared DoPL-D scores between groups of relatively older versus younger siblings using a t-test.

2.8.1. Cooperative Tetris Task analyses

Spearman's rank correlation was computed to assess the relationship between the total number of cleared lines and feedback between players. Moreover, in order to study the relationship between leadership role during the game (initiator of the actions) and dominance, bivariate correlates were calculated between the mean number of times the participant initiate the actions with the DoPL-D score, and dominance ICG score. Spearman's correlation was used to explore the relationship between dominance and the leadership question (corrected significance level α = 0.016). Finally, correlation analyses were performed between the total number of cleared lines and perception of success, shared mental representation, and preference to play as a team (corrected significance level α = 0.016).

As exploratory analyses, between-group comparisons using one-way ANOVAs for the task-related questions about the degree of enjoyment, difficulty, and previous experience with the Tetris game were calculated. Also, performance (number of total cleared lines) was compared between brothers and sisters using a t-test.

2.8.2. Interactive Chicken Game analyses

Furthermore, dominance (DoPL-D and ICG) scores were correlated with the motivation question and strategy questions to explore the relationship between dominance and motivation questions at individual level. Due to the correction for multiple comparisons,

the corrected significance level was $\alpha = 0.0025$. Lastly, an independent-sample t-test was performed to compare the condition outcomes between brother and sister pairs.

3. Supplementary Results

3.1. Questionnaires Correlations

Supplementary Table S6. Correlations between Questionnaire Scores *(N* = 56*)*

Variable	1	2	3	4	5	6	7	8	9	10	11	12
1. DoPL Dominance	–											
2. DoPL Prestige	0.27	–										
3. DoPL Leadership	0.58*	0.31	–									
4. RSPQ DL	0.37	0.02	0.70*	–								
5. RSPQ CB	-0.15	0.24	0.03	0.00	–							
6. RSPQ RSA	0.24	0.07	0.25	0.31	-0.08	–						
7. Mach-IV	0.51*	0.12	0.27	0.21	-0.34	0.53*	–					
8. HCA	0.46*	0.27	0.48*	0.48*	-0.27	0.46*	0.52*	–				
9. PDCA	0.18	0.27	0.16	0.21	0.08	-0.02	-0.24	0.07	–			
10. PSPS[1]	0.24	0.17	0.28	0.30	0.18	0.04	-0.18	-0.06	0.35	–		
11. STQ Criticism	0.49*	0.08	0.40	0.35	0.06	0.16	0.28	0.24	0.12	0.14	–	
12. STQ Apathy	0.36	0.05	0.15	0.16	-0.20	0.20	0.32	0.27	0.19	0.01	0.22	–
13. STQ Competition	0.48*	0.20	0.24	0.21	-0.19	0.14	0.20	0.41	0.19	0.28	0.56*	0.39

Note. *Significant after Bonferroni correction (corrected α = 0.0006), [1]Spearman correlation (others are calculated using Pearson correlation). DoPL = Dominance, Prestige, Leadership scale, RSPQ = Rank Style with Peers Questionnaire, DL = Dominant Leadership, CB = Coalition Building, RSA = Ruthless Self-Advancement, Mach-IV = Machiavellianism scale, HCA = Hypercompetitive Attitude scale, PDCA = Personal Development Competitive Attitude Scale, PSPS = Personal Sense of Power Scale, STQ = Sibling Type Questionnaire.

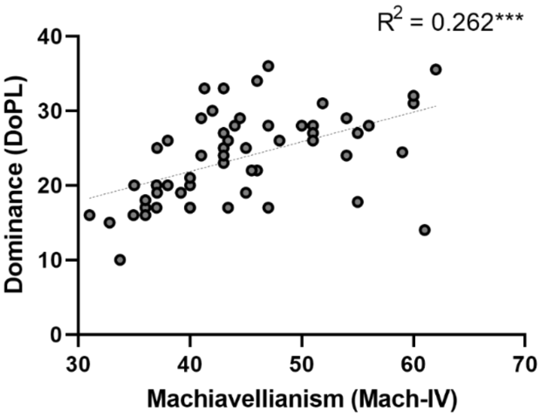

Figure S4. Scatter plot representing the relationship between scores on the Dominance subscale (DoPL-D) and the Machiavellianism scale (Mach-IV). *N* = 56, ***p < 0.001.

The comparison of the dominance scores (DoPL) between younger (*M* = 23.53, *SD* = 5.72) and older siblings (*M* = 23.41, *SD* = 6.88) was not significant ($t(46) = -0.096$, $p = 0.924$).

Supplementary Table S7. Sociodemographic characteristics of individual participants by clusters *(N* = 56*)*

Characteristic	Total *N* = 56		Cluster LL *n* = 14		Cluster LH *n* = 26		Cluster HH *n* = 16		Pearson's chi-squared test			Fisher's exact test
	N	%	*n*	%	*n*	%	*n*	%	X^2	*df*	*p*	*P*
**Marital status**									3.25[a]	6	0.777	0.900
Single	19	33.93	6	42.86	8	30.77	5	31.25				
In a relationship	32	57.14	8	57.14	15	57.69	9	56.25				
Married	4	7.14			2	7.69	2	12.50				
Not specified or missing data	1	1.79			1	3.85						
**Parents' marital status**									11.56[a]	8	0.172	0.120
Single	2	3.57					2	12.50				
Married to first spouse	35	62.50	14	100.00	13	50.00	8	50.00				
Divorced and remarried	4	7.14			2	7.69	2	12.50				
Other	4	7.14			2	7.69	2	12.50				
Not specified or missing data	11	19.65			9	34.62	2	12.50				
**Employment**									3.61[a]	6	0.729	0.781
Student	30	53.57	10	71.43	13	50.00	7	43.75				
Unemployed/seeking work	4	7.14	1	7.14	1	3.85	2	12.50				
Part-time employed	3	5.36			2	7.69	1	6.25				
Full-time employed	12	21.43	3	21.43	4	15.38	5	31.25				
Not specified or missing data	7	12.50			6	23.08	1	6.25				
**Highest educational level**									7.28[a]	8	0.507	0.615
General education school leaving certificate after grade 9	1	1.79			1	3.85						
General education school leaving certificate after grade 10	5	8.93			4	15.38	1	6.25				
Completed vocational training	4	7.14			3	11.54	1	6.25				
Higher education entrance qualification to study at a University of Applied Sciences	20	35.71	5	35.71	9	34.62	6	37.50				
General higher education entrance qualification	26	46.43	9	64.29	9	34.62	8	50.00				
**Monthly household budget**									12.70[a]	10	0.241	0.216

< 1,050 €	18	32.14	6	42.86	9	34.62	3	18.75
1,050 € to 1,410 €	7	12.50	3	21.43	2	7.69	2	12.50
1,410 € to 2,640 €	11	19.64	2	14.29	7	26.92	2	12.50
2,640 € to 4,400 €	7	12.50	2	14.29	1	3.85	4	25.00
> 4,400 €	2	3.57			1	3.85	1	6.25
Not specified or missing data	11	19.64	1	7.14	6	23.08	4	25.00

Note. [a] More than 20% of cells have an expected count of less than 5 and, therefore, Fisher's exact test should be considered instead[71]. LL = both low in dominance, LH = one low in dominance, and one high in dominance, and HH = both high in dominance. No significant differences were found in any demographic variables between clusters.

Supplementary Table S8. Personality characteristics of each cluster and total pairs.

	Total		Cluster LL		Cluster LH		Cluster HH		Statistics				Post-hoc tests		
	N = 28		n = 7		n = 13		n = 8						LL – LH	LL– HH	LH – HH
	M	*SD*	*M*	*SD*	*M*	*SD*	*M*	*SD*	F(2,25)	η_p^2	X^2(2 N = 28)	*p*	*p*	*p*	*p*
**DoPL**	34.61	4.49	30.50	2.32	34.25	3.96	38.81	3.00	11.49	0.48		**< 0.001**	0.077	**< 0.001**	**0.018**
Dominance	23.73	4.59	20.00	3.14	22.24	2.94	29.41	1.72	26.16	0.68		**< 0.001**	0.271	**< 0.001**	**< 0.001**
Dominance difference									21.60	0.63		**< 0.001**	**< 0.001**	0.171	**< 0.001**
Prestige	42.11	6.35	40.29	3.77	41.50	7.76	44.70	5.32	1.02	0.08		0.376			
Leadership	38.00	6.56	31.21	5.11	39.00	4.90	42.31	5.78	8.92	0.42		**0.001**	**0.011**	**0.001**	0.508
**Mach-IV**	2.48	0.36	2.29	0.31	2.38	0.32	2.82	0.25	7.26	0.37		**0.003**	1.000	0.007	0.009
**HCA**	64.26	8.07	56.46	4.65	65.20	7.90	69.55	5.58	7.55	0.38		**0.003**	**0.028**	**0.002**	0.468
**PDCA**	52.71	8.16	47.43	6.55	55.08	7.94	53.50	8.56	2.24	0.15		0.128			
**RSPQ**															
DL	18.52	3.38	14.36	2.94	19.81	2.34	20.06	2.04	13.78	0.52		**< 0.001**	**< 0.001**	**< 0.001**	1.000
CB	28.63	2.76	28.07	2.56	29.15	3.30	28.25	2.05	0.43	0.03		0.653			
RSA[a]	11.27	3.18	7.93	2.13	12.00	3.24	13.00	1.20	14.87[a]	0.40		**< 0.001**	**0.010**	**0.001**	0.583
**STQ**															
Mutuality[b]	49.46	7.24	55.07	4.23	46.23	8.12	49.81	4.75	5.48[b]	0.25		**0.015**	**0.013**	0.097	0.426

Competition	12.39	2.88	10.21	4.15	12.77	2.22	13.69	1.36	3.45	0.22		**0.048**	0.151	0.054	1.000
Criticism	24.46	6.78	20.07	4.25	24.73	7.92	27.88	4.63	2.83	0.19		0.078	0.392	0.077	0.845
Apathy	18.63	3.55	15.57	1.90	19.15	3.72	20.44	2.87	4.85	0.28		**0.017**	0.066	**0.018**	1.000
Longing	13.18	3.01	14.07	3.55	12.54	3.02	13.44	2.64	0.61	0.05		0.550	0.855	1.000	1.000
**PSPS**[c]	4.33	0.53	3.84	0.65	4.48	0.44	4.51	0.28			6.57	**0.037**	0.064	0.071	1.000

Note. All questionnaire results were computed as pair mean scores from both siblings' individual scores. [a] F-Welch (2,14.24) value calculated using Welch and Games-Howell tests, [b] F-Welch (2,16.18) value calculated using Welch and Games-Howell tests, [c] calculated using Kruskal-Wallis test. LL = both low in dominance, LH = one low in dominance, and one high in dominance, HH = both high in dominance, DoPL = Dominance, Prestige, Leadership scale, Mach-IV = Machiavellianism scale, HCA = Hypercompetitive Attitude scale, PDCA = Personal Development Competitive Attitude Scale, RSPQ = Rank Style with Peers Questionnaire, DL = Dominant Leadership, CB = Coalition Building, RSA = Ruthless Self-Advancement, STQ = Sibling Type Questionnaire, PSPS = Personal Sense of Power Scale. Statistically significant values are shown in bold.

3.2. Cooperative Tetris Task Correlations

A more positive feedback about their sibling's performance correlated positively with the total number of cleared lines ($r = 0.33$, $p = 0.012$). The correlation between initiator of action and DoPL dominance scores ($p = 0.700$), DoPL leadership scores ($p = 0.150$), and leader question ($p = 0.208$) were not significant. There was a significant positive correlation between initiator of action in the easy and difficult blocks ($r = 0.79$, $p < 0.0001$). Finally, there was a significant positive correlation between total number of cleared lines and success perception ($r = 0.38$, $p = 0.007$), as well as between total number of cleared lines and shared mental representation ($r = -0.48$, $p < 0.001$). There was no significant correlation between the total number of cleared lines and preference to play as a team rather than alone ($p = 0.736$). Clusters did not differ in enjoyment ($F(2,25) = 1.180$, $p = 0.324$), ($M = 3.96$, $SD = 0.52$), difficulty perception ($F(2,25) = 0.375$, $p = 0.490$), ($M = 2.29$, $SD = 0.74$), or previous Tetris experience ($F(2,25) = 0.021$, $p = 0.979$), ($M = 2.86$, $SD = 0.86$).

3.3. Interactive Chicken Game pair representative behaviors

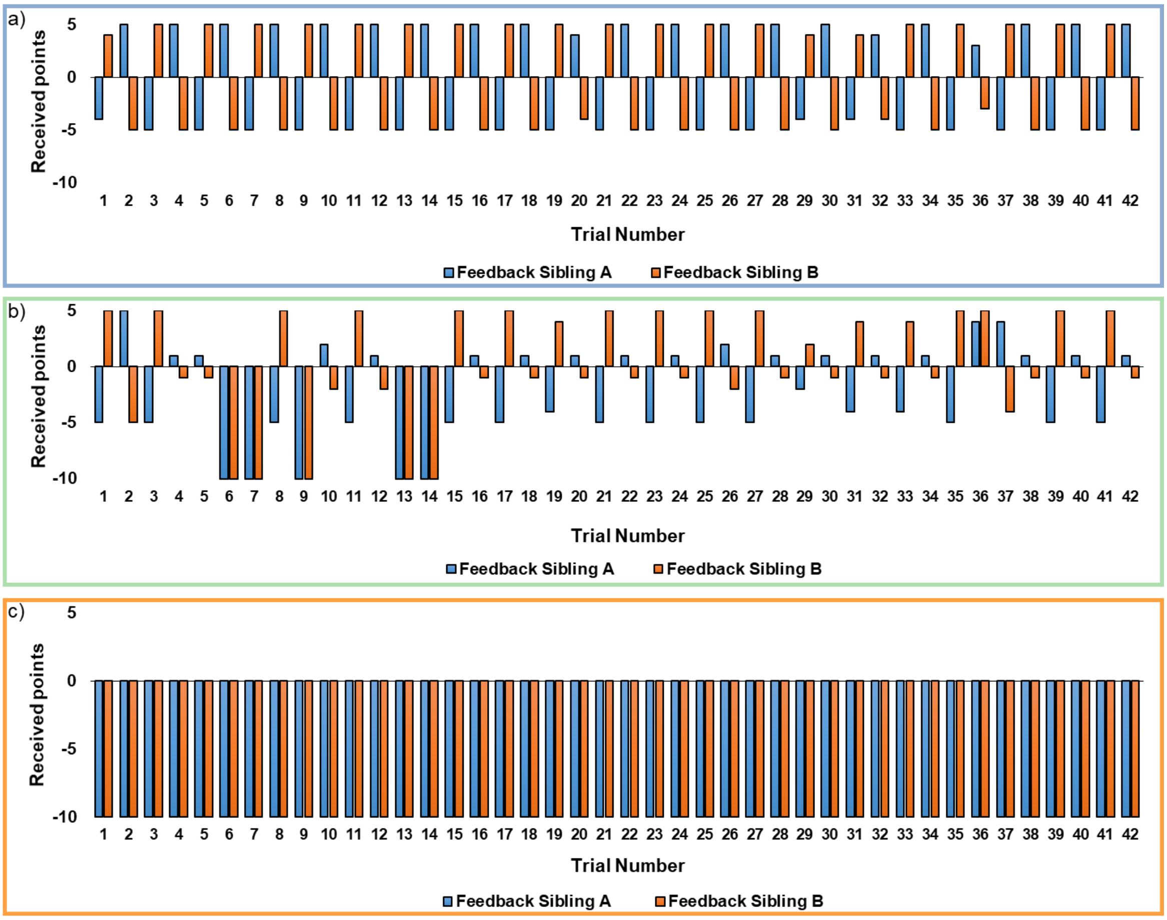

Figure S5. Bar charts depicting different representative behaviors during the Interactive Chicken Game task. Feedbacks (negative numbers equal losses, while positive numbers equal gains) received by each participant per trial are plotted. a) Example of a turn-taking strategy of a pair consisting of two individuals with low dominance. b) Example of a hetereogeneous pair including one individual with high dominance (sibling B, represented in orange) and one with low dominance (sibling A, represented in blue) who showed a more subordinate behavior turning more times and letting the other one gain more points. c) Example of both individuals with high dominance being all trials crashes.

3.4. Interactive Chicken Game performance

Supplementary Table S9. Interactive Chicken Game task performance.

	Cluster LL n = 6		Cluster LH n = 12		Cluster HH n = 8		Statistics				Post-hoc tests		
											LL–LH	LL–HH	LH–HH
	M	*SD*	*M*	*SD*	*M*	*SD*	$F(2,24)$	η_p^2	$X^2(2\ N = 26)^{1,2}$	*P*	*p*	*p*	*p*
Both crash	9.17	7.68	13.08	10.10	26.13	10.01	6.52	0.36		**0.006**	1.000	**0.010**	**0.020**
Both turn[a]	1.33	1.97	2.50	3.75	1.50	1.85			0.535	0.765			
One-turning	31.50	7.79	26.42	9.30	14.38	9.01	7.25	0.39		**0.004**	0.699	**0.005**	**0.021**
Turn-taking rounds	20.58	12.20	12.29	11.98	3.38	4.88			10.85	**0.004**	0.259	**0.003**	0.309
Dominance ICG score	179.17	151.18	240.58	216.40	507.75	211.22	5.78	0.33		**0.009**	1.000	**0.019**	**0.025**
Total ICG score	-187.50	156.07	-282.75	193.66	-537.25	190.19	7.16	0.38		**0.004**	0.626	**0.006**	**0.019**
Total ICG score difference[a]	26.83	22.30	33.25	29.62	23.00	27.32			0.686	0.710			

Note: ICG = Interactive Chicken Game, LL = both low in dominance, LH = one low in dominance, and one high in dominance, HH = both high in dominance. [a] variables calculated with the Kruskal-Wallis test. Statistically significant values are shown in bold.

3.5. Interactive Chicken Game Correlations

A significant positive correlation was found between dominance ICG scores (see Table S3 for score description) and the perception of the participant that their sibling behaved aggressively (see Table S5 ‘Sibling aggressive’) during the game (r = 0.41, p = 0.002). A positive correlation between dominance ICG scores and participants referring to themselves as behaving dominant during the game (r = 0.36, p = 0.010). A negative correlation between dominance ICG scores and participants referring to themselves as behaving subordinates during the game (r = -0.44, p = 0.001). Perception of fairness was negatively correlated with dominance ICG scores (r = -0.28, p = 0.042). Perception of

cooperation was negatively associated with dominance ICG score (r = -0.67, p < 0.0001), and dominance scores (DoPL) (r = -0.34, p = 0.014). Only the negative relationship between Dominance ICG score and submissive and perception of cooperation strategies survived after multiple comparison corrections. The motivation questions and other strategies were not significantly correlated with any dominance score (p > 0.05). Dominance ICG scores, and dominance scores (DoPL) had a significant positive correlation (r = 0.38, p = 0.006). All these findings serve as exploratory results.

3.6. Sex Differences

Supplementary Table S10. T-test results comparing brother and sister pairs on various measures.

	Females (n = 15 pairs)[c]		Males (n = 13 pairs)[c]		Statistics		
	M	*SD*	*M*	*SD*	*df*	*t*	*p*
**CoTT**							
Total success[a]	82.20	24.37	91.23	19.34	26	1.07	0.293
**ICG**							
Mutual defection (DD)[a]	10.93	8.19	22.33	12.08	24	2.85	**0.009**
One-turning (TD, DT)[a]	28.35	8.77	18.66	11.14	24	-2.48	**0.020**
Turn-taking strategy[a]	15.50	13.22	6.75	8.17	24	-1.98	0.059
Dominance ICG score[a]	197.43	163.45	444.69	241.36	24	2.94	**0.007**
**STQ**							
Mutuality[b]	53.23	6.22	45.12	7.32	54	-4.49	**< 0.001**
Competition[b]	12.03	3.71	12.81	4.19	54	0.72	0.475
Criticism[b]	25.00	8.45	23.85	7.73	54	-.053	0.598
Apathy[b]	17.63	4.04	19.77	4.19	54	1.94	0.058
Longing[b]	14.47	3.18	11.69	2.75	54	-3.46	**0.001**
**DoPL**							
Dominance[b]	23.49	6.27	24.01	5.95	54	0.32	0.751

Note. [a]sibling pair score, [b]individual score. Statistically significant values are shown in bold, [c] ICG sample size is females n = 14, males n = 12. CoTT = Cooperation Tetris task, ICG = Interactive Chicken game, STQ = Sibling Type Questionnaire, DoPL = Dominance, Prestige, Leadership scale.

Brother and sister pairs did not significantly differ in success. Consistent with our hypothesis, brothers were more dominant than sisters, as they had more crashes and a higher dominance score, while sisters had only one-turningmore frequently. This is supported by previous literature showing that brothers have more sibling conflicts[1,2], as well as having more power struggles[3]. However, there is also support for more frequent

and intense conflicts between sisters[4], or absence of differences between brothers and sisters[5,6]. Sisters have a more supportive, affective, similar, and close relationship, in line with previous literature describing sister-sister relationships as more emotionally close than between brothers[7,8]. Given that brother and sister pairs did not significantly differ in their dominance score (DoPL), we interpret that the differences in dominance behavior in the task are more likely due to differences in their sibling relationship.

References

1. Salmon, C. & Hehman, J. Evolutionary Perspectives on the Nature of Sibling Conflict: the Impact of Sex, Relatedness, and Co-residence. *Evol. Psychol. Sci.* **1**, 123–129. https://doi.org/10.1007/s40806-015-0013-9 (2015).
2. Straus, M. A., Gelles, R. J. & Stienmetz, S. K. *Behind Closed Doors: Violence in the American Family*. https://doi.org/10.4324/9781351298681 (Routledge, 2006).
3. Lindell, A. K. & Campione-Barr, N. Relative Power in Sibling Relationships Across Adolescence: Relative Power In Sibling Relationships Across Adolescence. *New Dir. Child Adolesc. Dev.* **2017**, 49–66. https://doi.org/10.1002/cad.20201 (2017).
4. Salmon, C. A. & Hehman, J. A. Good Friends, Better Enemies? The Effects of Sibling Sex, Co-Residence, and Relatedness on Sibling Conflict and Cooperation. *Evol. Psychol. Sci.* **7**, 327–337. https://doi.org/10.1007/s40806-021-00292-y (2021).
5. Hehman, J. A., Burch, R. L. & Salmon, C. A. Sibling Conflict and Closeness: The Effects of Sex, Number of Siblings, Relatedness, Parental Resemblance and Investment. *Evol. Psychol. Sci.* https://doi.org/10.1007/s40806-022-00353-w (2023).
6. Khan, R., Brewer, G. & Archer, J. Genetic relatedness, emotional closeness and physical aggression: A comparison of full and half sibling experiences. *Eur. J. Psychol.* **16**, 167–185. https://doi.org/10.5964/ejop.v16i1.1620 (2020).

7. Tanskanen, A. O. & Danielsbacka, M. Relationship Quality Among Half Siblings: the Role of Childhood Co-residence. *Evol. Psychol. Sci.* **5**, 13–21. https://doi.org/10.1007/s40806-018-0161-9 (2019).

8. Stocker, C. M. *et al.* Sibling relationships in older adulthood: Links with loneliness and well-being. *J. Fam. Psychol.* **34**, 175–185. https://doi.org/10.1037/fam0000586 (2020).
